# Trans-Influence-Driven Coordination Plasticity in Carbazolide NNN Pincer Nickel(II) Complexes

**DOI:** 10.1021/acs.inorgchem.6c02664

**Published:** 2026-08-22

**Authors:** Jun-Yang Ye, Shu-Chi Liao, Matthias Zeller, Wei-Tsung Lee

**Affiliations:** † Department of Chemistry, 34911 National Central University, Taoyuan 32001, Taiwan; ‡ Department of Chemistry, 8522 Purdue University, West Lafayette, Indiana 47907, United States

## Abstract

Carbazolide-based NNN pincer ligands impart a strong electronic trans influence at nickel­(II) centers, leading to unusual coordination behavior. A series of Ni­(II) complexes supported by Cz^*t*Bu^(Pyr^H^)_2_^–^ reveals pronounced coordination plasticity depending on the ancillary ligand and solvent environment. Halide complexes preferentially form solvated octahedral species in coordinating media and exhibit partial ionization of the Ni–X bond, as supported by salt-metathesis experiments and ionic conductivity measurements. Oxygen-donor ligands promote μ-alkoxide dimer formation with temperature-dependent solution dynamics, while strongly donating ligands such as CF_3_^–^ and N­(SiMe_3_)_2_^–^ stabilize classical square-planar geometries. The fluoride complex displays solvent-dependent speciation, with hydrogen-bonding interactions in CHCl_3_ stabilizing a monomeric square-planar structure. Comparison with diphenylamide analogues confirms that the rigid and strongly donating carbazolide backbone is responsible for this behavior. These results demonstrate how backbone electronics can modulate Ni–ligand lability and coordination geometry in pincer complexes.

## Graphical abstract

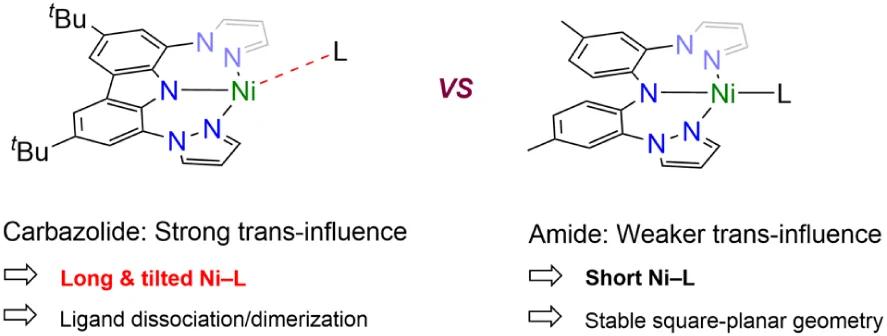

## Introduction

The development of pincer-type ligands has significantly impacted transition metal chemistry, providing a versatile platform for stabilizing reactive intermediates and facilitating challenging catalytic transformations such as C–H activation, cross-coupling, and small molecule activation.^1–7^ Among the diverse array of pincer architectures, those featuring a central anionic donor, specifically NNN-type frameworks, are highly valued for their strong σ-donating ability and the meridional coordination environment they enforce upon the metal center.^8^ In particular, the “amido effect” of these ligands has been shown to dramatically influence the electronic properties and reactivity of late transition metals,^9^ often leading to unconventional coordination numbers or spin states.^10,11^

Within this class, the carbazole-based NNN framework has emerged as a particularly effective scaffold.^3,12^ Its inherent rigidity, extended π-system, and planar geometry allow for precise electronic tuning while maintaining a robust ligand environment.^13–18^ We recently reported a series of sterically encumbered Ni­(II) complexes supported by the Cz^*t*Bu^(Pyr^*i*Pr^)_2_^–^ ligand (where Pyr^*i*Pr^ = 3-isopropylpyrazole). In that system, the significant steric bulk of the isopropyl groups on the pyrazole arms prevented the nickel center from adopting a square planar configuration, instead enforcing a rare and distorted “seesaw” geometry (Chart [Fig chart1]).^3^ These complexes exhibited fascinating coordination dynamics, including reversible solvent binding to form five-coordinate adducts and LMCT-driven thermochromic behavior. While these findings highlighted the structural effect of the NNN-carbazole manifold, the overwhelming steric protection likely masked the intrinsic electronic preferences of the system.

**1 chart1:**
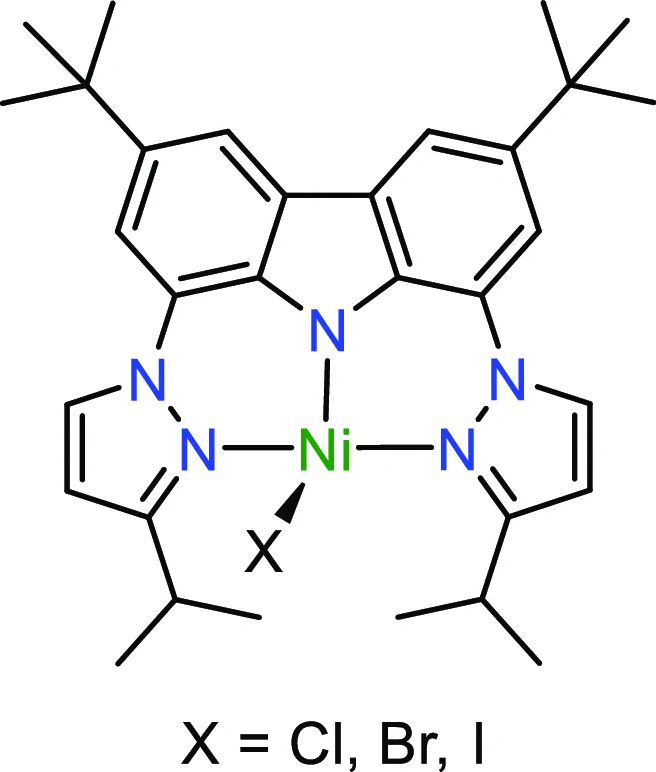
Chemical Structure of [Cz^*t*Bu^(Pyr^*i*Pr^)_2_NiX] (X = Cl, Br, I)

To isolate the intrinsic electronic contribution of the carbazolide backbone from steric effects, we prepared a less encumbered ligand variant bearing 3-unsubstituted pyrazoles, Cz^*t*Bu^(Pyr^H^)_2_^–^. Unexpectedly, the targeted square-planar halide complexes proved elusive. We hypothesized that the strong σ- and π-donating ability of the central carbazolide nitrogen exerts an unusually potent trans influence, labilizing the trans Ni–X bond and promoting solvation, ionization, or dimerization. A systematic study of more than ten complexes spanning the spectrochemical series, supported by X-ray crystallography, variable-temperature NMR, ionic conductivity, and comparison with flexible diphenylamide analogues, reveals that backbone-driven electronic effects alone can override the classical square-planar preference of *d* ^8^ Ni­(II), giving rise to pronounced coordination plasticity.

## Results and Discussion

### Synthesis and Instability of Nickel­(II) Halide Complexes

Initial attempts to prepare the square-planar halide complexes [Cz^*t*Bu^(Pyr^H^)_2_NiX] (X = Cl, Br, I) via deprotonation of HCz^*t*Bu^(Pyr^H^)_2_ with LDA or KO^*t*^Bu followed by transmetalation with NiX_2_(DME) or NiX_2_(THF)_1.5_ yielded brown solutions in THF. However, removal of THF or exposure to noncoordinating solvents (toluene, CH_2_Cl_2_, CHCl_3_) led to rapid disproportionation to the homoleptic species [(Cz^*t*Bu^(Pyr^H^)_2_)_2_Ni] and NiX_2_ (Figure S12).^19^ This instability suggests that the target four-coordinate square planar complex [Cz^*t*Bu^(Pyr^H^)_2_NiX] is electronically unfavorable in the absence of coordinating solvents.

To circumvent this decomposition in noncoordinating solvents, reaction in hot THF (70 °C), followed by filtration and crystallization, allowed for the isolation of the paramagnetic, six-coordinate adduct [Cz^*t*Bu^(Pyr^H^)_2_NiBr­(THF)_2_] in high yield. ^1^H NMR monitoring confirmed that the presence of coordinating solvent is essential for the integrity of the complex (Scheme 1). Removal of THF under vacuum results in an immediate color change, consistent with desolvation of the octahedral precursor. Although repeated attempts to crystallographically characterize the resulting intermediate were unsuccessful because of its rapid decomposition in noncoordinating media, its markedly reduced stability relative to solutions containing THF suggests that solvent coordination plays a crucial role in stabilizing the complex.

**Scheme 1:**
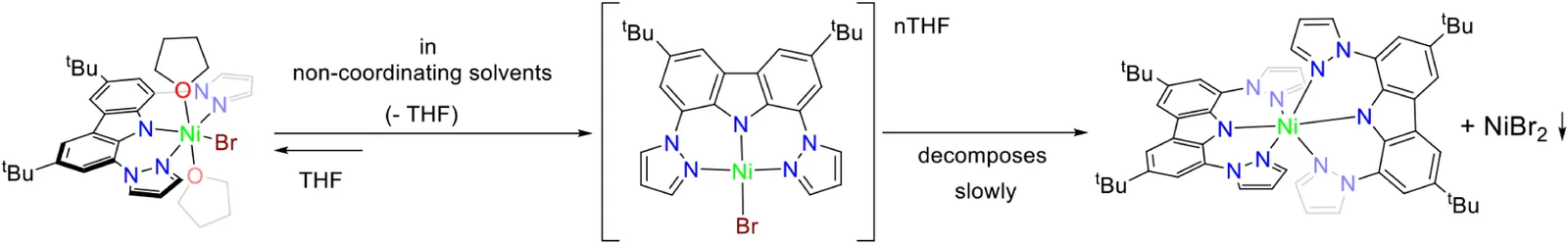
Synthesis and Solvent-Dependent Stability of [Cz^*t*Bu^(Pyr^H^)_2_NiBr­(THF)_*x*_]

The chloride complex, [Cz^*t*Bu^(Pyr^H^)_2_NiCl­(THF)_2_], exhibits similar paramagnetic behavior (Figure S13) in THF-*d*_8_ but displays greater stability than the bromide analogue, showing no immediate decomposition in nonpolar media. Conversely, the iodide analogue is highly unstable. While it appears to form a similar solvated structure in THF (based on paramagnetic NMR features), it decomposes rapidly into [(Cz^*t*Bu^(Pyr^H^)_2_)_2_Ni] and NiI_2_ upon exposure to noncoordinating solvents (Figure 1) or even upon prolonged standing in THF, precluding single-crystal isolation. The visual instability shown in Figure 1 is consistent with the lability of the Ni–X bond induced by the strongly donating carbazolide framework.

**Figure 1:**
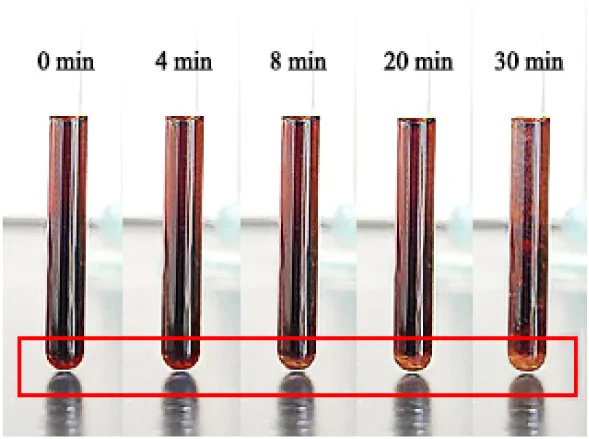
Time-lapse photographs showing the degradation of [Cz^*t*Bu^(Pyr^H^)_2_NiI] in CH_2_Cl_2_ at room temperature. The initially clear red solution gradually decomposes over 30 min, forming a beige suspension and precipitated NiI_2_. The images are original photographs taken by the authors and are shown as standalone stills from the accompanying reaction video.^20^

### Structural Evidence of the Trans Influence and Cationic Dissociation

Single-crystal X-ray diffraction of [Cz^*t*Bu^(Pyr^H^)_2_NiCl­(THF)_2_] and [Cz^*t*Bu^(Pyr^H^)_2_NiBr­(THF)_2_] revealed octahedral geometries with two axial THF molecules (Figure 2). A striking feature of these structures is the elongation of the Ni–X bonds (Table 1). The Ni–Br bond length is 2.5963(5)/2.5672(8) Å, and the Ni–Cl bond is similarly elongated (2.418(3)/2.415(2) Å). These values are unusually long, pointing to a potent trans influence exerted by the central carbazolide nitrogen. We hypothesize that the strong σ- and π-donating ability of the carbazolide backbone increases electron density at the nickel center, thereby labilizing the trans-disposed halide. This labilization likely promotes ionization in solution. High-resolution MALDI-TOF mass spectrometry of the halide complexes revealed a dominant peak corresponding to the cationic [Cz^*t*Bu^(Pyr^H^)_2_Ni]^+^ fragment. Interestingly, dissolving [Cz^*t*Bu^(Pyr^H^)_2_NiBr] in MeCN-*d*_3_ (Figure S14), a stronger field solvent/ligand, resulted in a deep red solution with a paramagnetic ^1^H NMR spectrum closely resembling those of the THF-derived species (Figure S13, bottom), indicating the formation of a highly solvated MeCN adduct. While partial bromide dissociation is likely in view of the behavior observed in THF, the available data do not permit definitive assignment of the solution structure.

**Figure 2:**
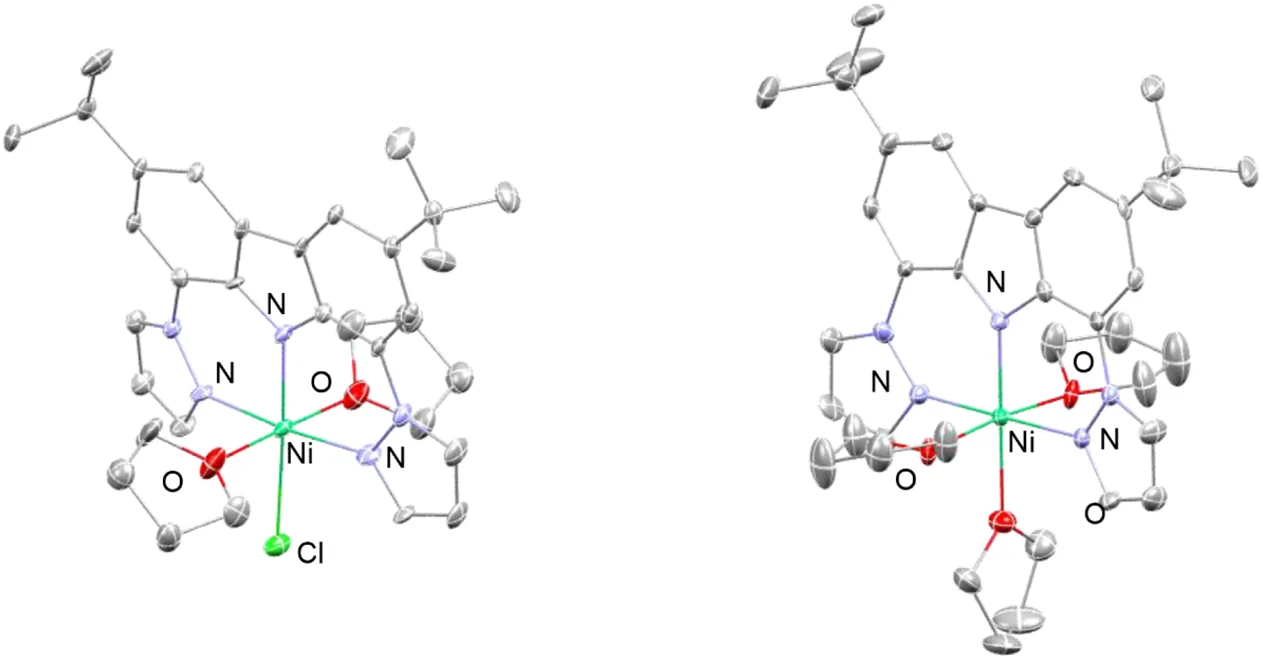
Molecular structure of [Cz^*t*Bu^(Pyr^H^)_2_NiCl­(THF)_2_] (left) and [Cz^*t*Bu^(Pyr^H^)_2_Ni­(THF)_3_]^+^ (right, anion is BAr^F^_4_^–^) with thermal ellipsoids at the 50% probability level. For [Cz^*t*Bu^(Pyr^H^)_2_NiCl­(THF)_2_], one of two crystallographic independent molecules is shown. Color code: N, blue; C, gray; Ni, teal; O, red; Cl, green. Hydrogen atoms and solvent molecules are omitted for clarity.

**Table 1:** Selected Bond Distances (Å) and Angles (°) for Nickel Halide Complexes

	Ni–X	N–Ni–X
Cz^*t*Bu^(Pyr^H^)_2_NiCl(THF)_2_	2.418(3)/2.415(2)	176.5(2)/175.9(2)
Dpa^Me^(Pyr^H^)_2_NiCl	2.205(1)/2.202(1)	175.5(1)/178.7(1)
Cz^*t*Bu^(Pyr^*i*Pr^)_2_NiCl^3^	2.2423(7)	129.44(7)
Cz^*t*Bu^(Pyr^H^)_2_NiBr(THF)_2_	2.5963(5)/2.5672(8)	175.67(8)/175.15(8)
Dpa^Me^(Pyr^H^)_2_NiBr	2.3528/2.3496	180.0
Cz^*t*Bu^(Pyr^*i*Pr^)_2_NiBr^3^	2.3807(5)	127.42(7)

Furthermore, [Cz^*t*Bu^(Pyr^H^)_2_NiBr­(THF)_2_] was treated with AgBAr^F^_4_ (BAr^F^_4_ = ([B­(C_6_H_3_-3,5-(CF_3_)_2_)_4_]^−^) in THF. The resulting species exhibited a ^1^H NMR spectrum similar to the neutral halide precursors (Figure S13). X-ray characterization confirmed the formation of [Cz^*t*Bu^(Pyr^H^)_2_Ni­(THF)_3_]­[BAr^F^_4_], a rare example of a Ni­(II) tris-THF solvated complex. The structural and spectroscopic similarity between the neutral halides in THF and this authentic cation strongly suggests that in coordinating media, the halides exist primarily as separated ion pairs, [Cz^*t*Bu^(Pyr^H^)_2_Ni­(THF)_3_]^+^[BAr^F^_4_]^−^, driven by the carbazolide-induced lability of the Ni–X bond.

To further evaluate this hypothesis, ionic conductivity (σ) measurements were performed by electrochemical impedance spectroscopy in 1 mM THF solutions. The nondissociative complexes, [Cz^*t*Bu^(Pyr^*i*Pr^)_2_NiBr] and [Cz^*t*Bu^(Pyr^H^)_2_Ni­(μ-OMe)]_2_ (vide infra) exhibited negligible conductivity with σ values of 0.16 and 0.29 μS/cm, respectively. In contrast, the neutral bromide [Cz^*t*Bu^(Pyr^H^)_2_NiBr­(THF)_2_] displayed a significantly higher conductivity of 1.96 μS/cm. Although this value remains substantially lower than those measured for the authentic ionic complex [Cz^*t*Bu^(Pyr^H^)_2_Ni­(THF)_3_]­[BAr^F^_4_] (21.2 μS/cm) and the standard electrolyte NBu_4_PF_6_ (9.54 μS/cm), it is approximately 1 order of magnitude greater than those of the nondissociative control complexes. These results indicate appreciable ionic character in solution and are consistent with partial ionization of the Ni–Br bond in coordinating media. Taken together with the isolation of the cationic complex [Cz^*t*Bu^(Pyr^H^)_2_Ni­(THF)_3_]^+^ and the close similarity of their solution NMR spectra, the conductivity data support an equilibrium between the neutral bromide complex and a solvent-stabilized cationic species in THF.

### Pseudohalides

Reaction with NaN_3_ yielded the azide complex [Cz^*t*Bu^(Pyr^H^)_2_NiN_3_], which is quite stable in various solvents, likely due to the stronger binding affinity of the azide anion preventing dissociation. Interestingly, the ^1^H NMR spectrum of [Cz^*t*Bu^(Pyr^H^)_2_NiN_3_] in THF-*d*_8_ (Figure S13) suggests a six-coordinate paramagnetic species, while in CDCl_3_, the signals appear in the diamagnetic region but exhibit significant broadening, particularly in the aromatic region. Evans method measurements confirm diamagnetism in solution, indicating that the line broadening likely arises from rapid solution dynamics or possible antiferromagnetic coupling in transient azido-bridged dimers.

### The Fluoride Anomaly and Hydrogen Bonding

The fluoride derivative, [Cz^*t*Bu^(Pyr^H^)_2_NiF], serves as a sensitive probe for the electronic influence of the secondary coordination sphere, exhibiting a solution profile that varies dramatically with the medium (Figures S13 and S15). In THF-*d*_8_, the complex is forest-green and paramagnetic. This indicates the coordination of axial THF molecules to form a high-spin octahedral species.

A transition toward the diamagnetic region is first observed in C_6_D_6_, though the resonances are exceptionally broad. Consistent with our observations for the alkoxide analogues (vide infra), this broadening likely reflects a rapid monomer–dimer equilibrium on the NMR time scale in noncoordinating media. “In the electron-rich environment imparted by the carbazolide backbone, the 16-electron square-planar monomer is thermodynamically disfavored, promoting association into a fluoride-bridged dimeric structure.

The stabilization of the monomeric low-spin state becomes more pronounced in chlorinated solvents as hydrogen-bonding interactions intervene. In CD_2_Cl_2_, the resonances remain in the diamagnetic region and begin to coalesce, though they remain broader than in CDCl_3_. We attribute this to a weak secondary-sphere interaction with the dichloromethane protons, which begins to interact with the fluoride ligand, mitigating the trans-influence and shifting the equilibrium toward the monomeric singlet state, albeit without fully locking the geometry.

In CDCl_3_, the spectrum sharpens into a well-resolved diamagnetic pattern characteristic of a square-planar Ni­(II) complex. The increased acidity of the chloroform proton enables a robust Ni–F···H–CCl_3_ hydrogen bond, confirmed crystallographically with an F···H distance of 2.034 Å (Figure 3). This interaction anchors the fluoride ligand and effectively withdraws electron density, suppressing the trans-repulsion from the carbazolide backbone and preventing the dimerization or structural fluctuations observed in C_6_D_6_ or THF-*d*_8_. This progression from a paramagnetic solvate to an associating equilibrium, and finally to a hydrogen-bond-stabilized monomer highlights the profound impact of the chemical environment on the stability of carbazolide-based pincers. Notably, despite the comparatively sharper ^1^H NMR spectrum in CDCl_3_, no ^19^F resonance was observed over a wide spectral window (δ = +1000 to −1000 ppm) at 298 K. However, upon cooling to −40 °C, a very broad resonance emerges at approximately −416 ppm (Figure S4 inset).^21^ This behavior is consistent with severe line broadening arising from residual exchange processes and the strong hydrogen-bonding interaction, to which the ^19^F nucleus is particularly sensitive. Although a fluoride-bridged dimer of this system could not be crystallographically characterized, a structurally related Ni–F analogue, [Cz^*t*Bu^(Pyr^*i*Pr^)_2_Ni­(μ-F)]_2_ (Figure S18), was found to adopt a μ-fluoride-bridged dimeric structure, supporting the assignment of a monomer–dimer equilibrium in solution.

**Figure 3:**
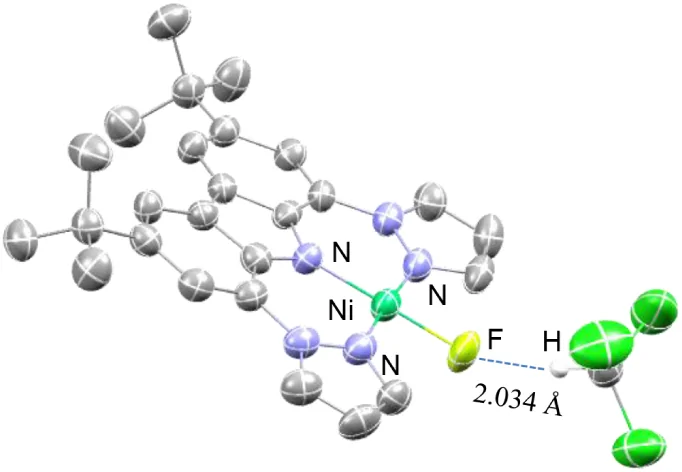
Molecular structure of [Cz^*t*Bu^(Pyr^H^)_2_NiF] with thermal ellipsoids at the 50% probability level. Color code: N, blue; C, gray; Ni, teal; F, yellow-green; Cl, green; H, white. All hydrogen atoms on the supporting ligand are omitted for clarity.

### Alkoxides and Monomer–Dimer Equilibrium

To probe the effect of oxygen donors, we attempted to synthesize the alkoxido derivatives [Cz^*t*Bu^(Pyr^H^)_2_NiOR] (R = Me, Bn). The ^1^H NMR spectra of these complexes in CDCl_3_ closely resemble that of the azido complex, showing broad resonances in the diamagnetic region. X-ray crystallography of the methoxido derivatives revealed dimeric structures, [Cz^*t*Bu^(Pyr^H^)_2_Ni­(μ-OR)]_2_, while the bulkier benzyloxido complex crystallized as a monomer (Figure 4). Despite the monomeric solid-state structure of [Cz^*t*Bu^(Pyr^H^)_2_NiOBn], its solution ^1^H NMR remains broad, showing a similar behavior to the methoxide analogue, suggesting a rapid monomer–dimer equilibrium on the NMR time scale. We attribute this behavior to the electron-rich carbazolide backbone, which destabilizes the coordinatively and electronically unsaturated 16-electron square-planar [Cz^*t*Bu^(Pyr^H^)_2_NiOR] monomer, favoring dimerization or solvation as pathways to a more stable, higher-coordinate species.

**Figure 4:**
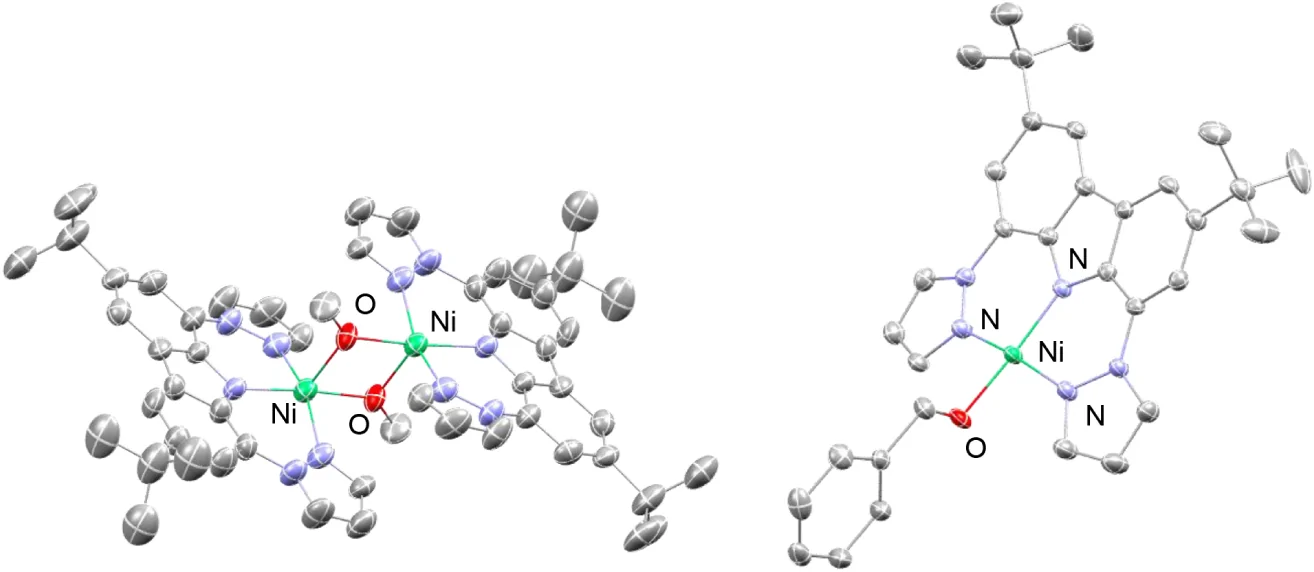
Molecular structure of [Cz^*t*Bu^(Pyr^H^)_2_Ni­(μ-OMe)]_2_ (left) and [Cz^*t*Bu^(Pyr^H^)_2_NiOBn] (right) with thermal ellipsoids at the 50% probability level. For [Cz^*t*Bu^(Pyr^H^)_2_Ni­(μ-OMe)]_2_, one of two crystallographically independent molecules is shown. Color code: N, blue; C, gray; Ni, teal; O, red. Hydrogen atoms and noncoordinating solvent are omitted for clarity.

To further probe this dynamic behavior, variable-temperature ^1^H NMR experiments were conducted (Figure 5). Upon cooling, the resonances become progressively sharper and more resolved, consistent with reduced dynamic exchange at low temperature. We attribute this sharpening to the increasing population of a diamagnetic, antiferromagnetically coupled μ-alkoxide-bridged dimer, consistent with the thermodynamics of an association equilibrium, in which enthalpically favorable Ni–O bond formation increasingly outweighs the entropic penalty of dimerization as temperature decreases. While we cannot completely exclude a minor contribution from reduced dynamic exchange within a persistent monomer population, a net increase in monomer population upon cooling would run counter to the expected thermodynamics of an enthalpy-driven association equilibrium, in which lower temperatures should favor the more associated (dimeric) state. Given the likelihood of multiple overlapping processes, the VT NMR data are discussed qualitatively and were not used to extract thermodynamic parameters.

**Figure 5:**
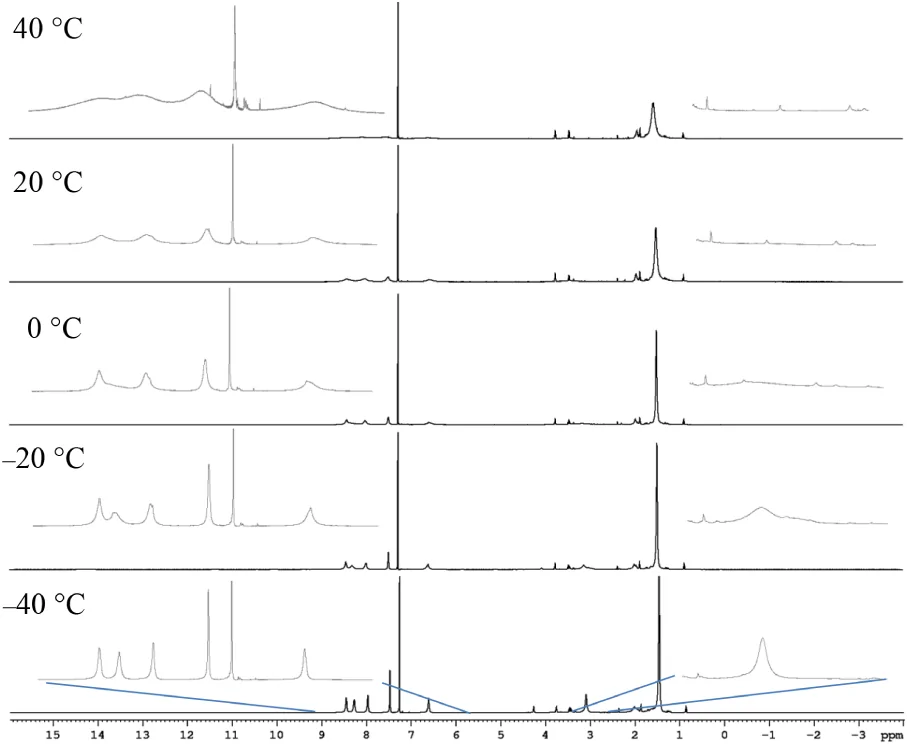
VT ^1^H NMR spectra (500 MHz, CDCl_3_) of [Cz^*t*Bu^(Pyr^H^)_2_Ni­(μ-OMe)]_2_ recorded between 40 and −40 °C. Insets show the expansion of resonances in the 9 to 6 ppm and 3.4 to 2.6 ppm regions.

### Stabilization via Strong-Field Ligands

Based on the observations above, we hypothesized that stable square planar geometries could only be enforced by ligands combining high ligand field strength with sufficient steric bulk. This was confirmed by the synthesis of [Cz^*t*Bu^(Pyr^H^)_2_NiN­(SiMe_3_)_2_] and [Cz^*t*Bu^(Pyr^H^)_2_Ni­(CF_3_)].

[Cz^*t*Bu^(Pyr^H^)_2_NiN­(SiMe_3_)_2_] appears as a classical diamagnetic, square planar complex with sharp ^1^H NMR signals in all tested solvents. Although amido ligands are considered as moderate ligand-field strength, the potential monomer–dimer equilibrium is mitigated due to the steric effect of the bulky N­(SiMe_3_)_2_^–^ group. The trifluoromethyl complex, [Cz^*t*Bu^(Pyr^H^)_2_Ni­(CF_3_)], featuring a very strong-field,^22^ organometallic carbon-donor was isolated as a stable, diamagnetic red solid. In contrast to [Cz^*t*Bu^(Pyr^H^)_2_NiF], a well-resolved ^19^F NMR resonance (−29 ppm) is observed for the CF_3_ ligand, consistent with a rigid and electronically well-defined coordination environment. Crystal structures of both complexes confirm distorted square planar geometries (Figure 6 and Table 2). These results definitively show that the strong trans influence and electronic donation of the carbazolide pincer require strong-field ancillary ligands to stabilize the low-spin *d* ^8^ configuration and suppress the tendency toward octahedral coordination or decomposition.

**Figure 6:**
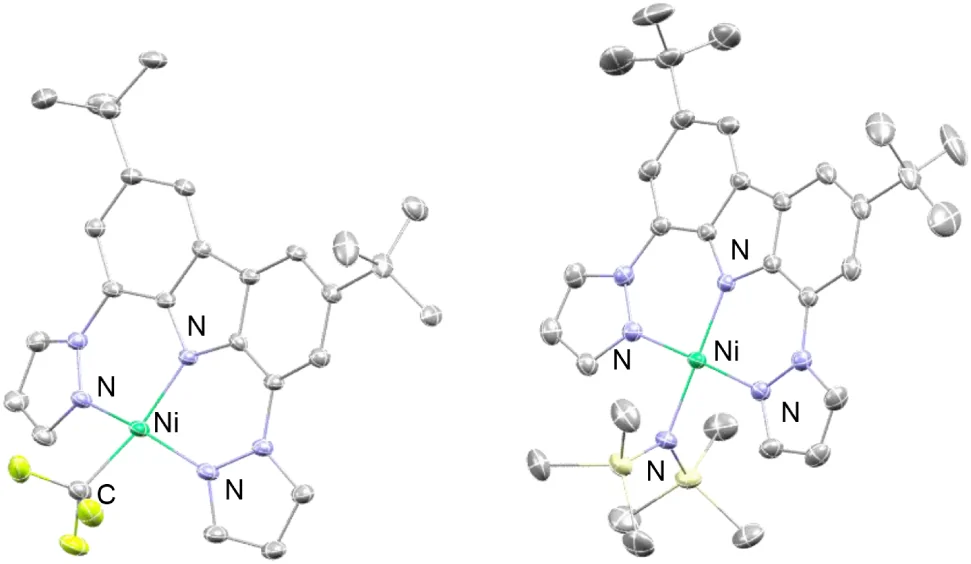
Molecular structure of [Cz^*t*Bu^(Pyr^H^)_2_Ni­(CF_3_)] (left) and [Cz^*t*Bu^(Pyr^H^)_2_NiN­(SiMe_3_)_2_] (right) with thermal ellipsoids at the 50% probability level. Color code: N, blue; C, gray; Ni, teal; F, yellow-green; Si, light-yellow. Hydrogen atoms and noncoordinating solvent are omitted for clarity.

**Table 2:** Selected Bond Distances (Å) and Angles (°) for Cz^*t*Bu^(Pyr^H^)_2_NiX

Cz^*t*Bu^(Pyr^H^)_2_NiX	Ni1–X1	N3_(Cz)_–Ni1–X1	τ_4_
X = F	1.827(3)	179.6(2)	0.03
X = OMe	1.999(5)	169.2(2)	-
X = OBn	1.854(3)	175.0(1)	0.10
X = CF_3_	1.927(3)	164.9(1)	0.24
X = N(SiMe_3_)_2_	1.930	180.0	0.01

### Discussion of Ligand Backbone Effects

To contextualize the coordination plasticity induced by the carbazolide backbone, we compared these systems with structurally related NNN-type pincer frameworks. The coordination geometry at the Ni­(II) center was quantified using the τ_4_ parameter, while the ∠N–Ni–X angle was used as a metric for trans influence, with increasing deviation from 180° indicating greater labilization of the Ni–X bond. As shown in Chart [Fig chart2], NNN pincer systems featuring central pyridine,^23–25^ amide,^26,27^ or pyrrolide^28^ donors typically adopt robust square-planar geometries in the presence of halide ligands. In these systems, the trans influence of the central donor is insufficient to significantly weaken the Ni–X bond or destabilize the square-planar manifold. In contrast, the strongly donating and rigid carbazolide backbone promotes substantial Ni–X labilization, accounting for the absence of well-defined square-planar Ni­(II) halide species in this ligand framework. This comparison is particularly relevant because, while NNN pincer architectures typically favor a low-spin square planar geometry, these specific Ni­(II) halide species have remained elusive in carbazole-based systems.

**2 chart2:**
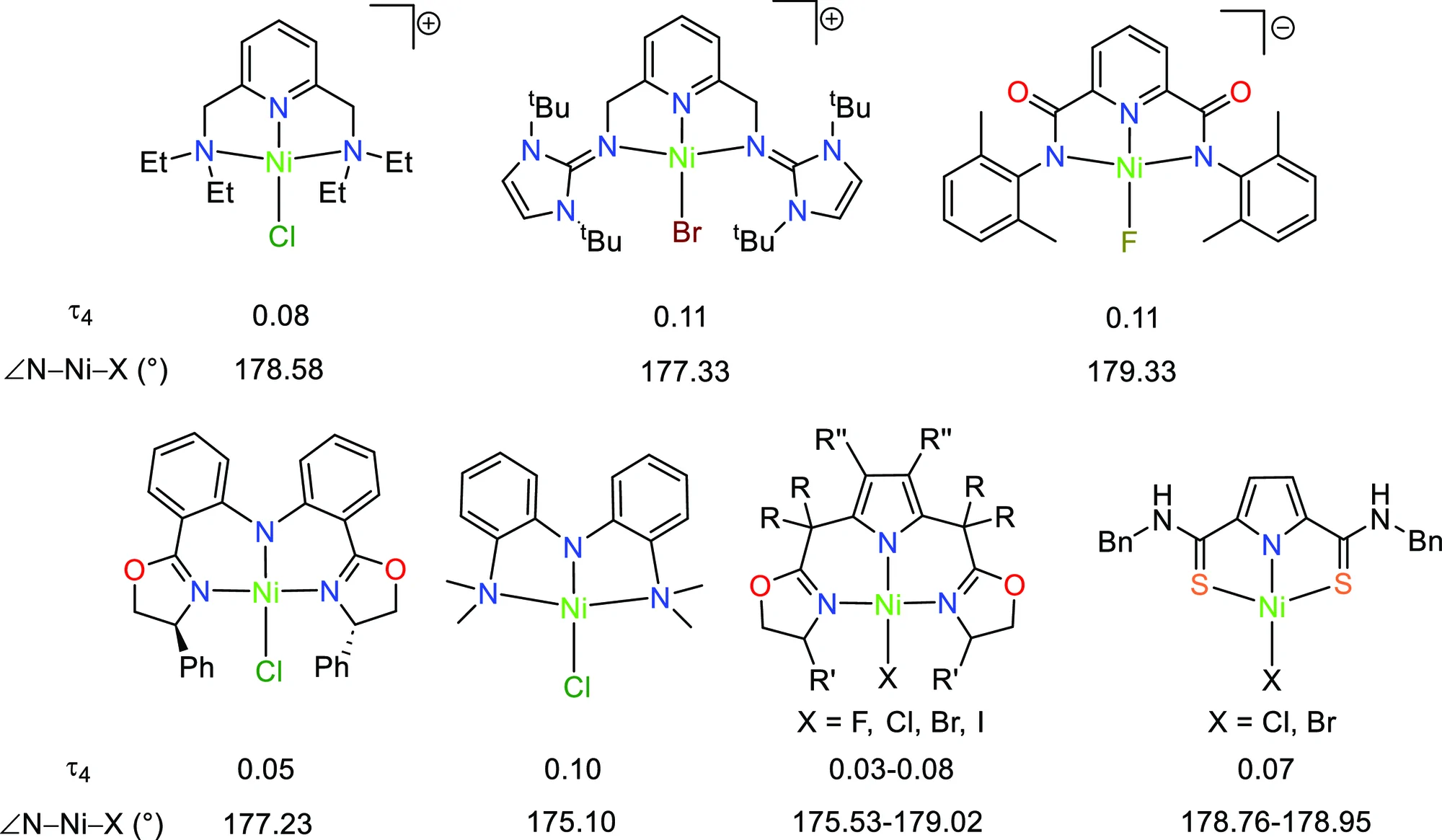
Pincer-Type Nickel­(II) Halide Complexes

To isolate the electronic influence of the carbazole backbone and determine if the coordination plasticity was a consequence of the pyrazole flanking arms, we synthesized and characterized a series of structural analogues featuring a central diphenylamide backbone [Dpa^Me^(Pyr^H^)_2_NiX] (X = Cl, Br, I). In stark contrast to the carbazolide series, these diphenylamide-based complexes were synthesized. Their solid-state structures consistently adopt four-coordinate square planar geometries, which is confirmed by the X-ray crystal structures (Figure 7), and all of which reside in the low-spin, diamagnetic manifold as evidenced by their sharp ^1^H NMR spectra (Figures S9–S11).

**Figure 7:**
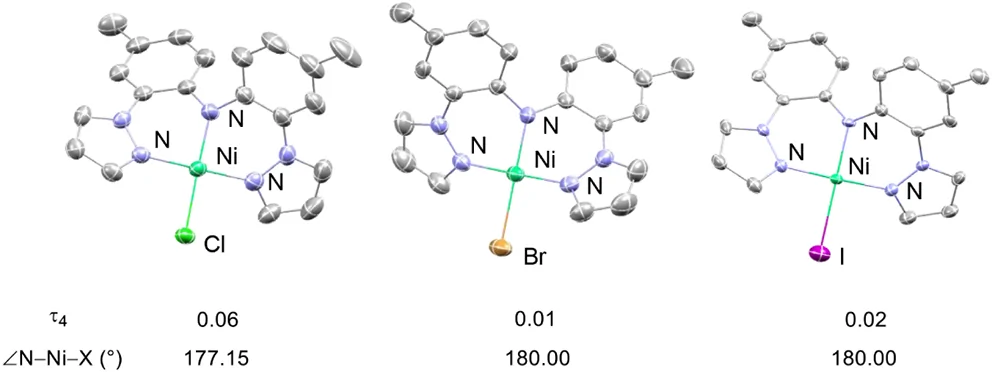
Molecular structure of [Dpa^Me^(Pyr^H^)_2_NiCl] (left), [Dpa^Me^(Pyr^H^)_2_NiBr] (middle), and [Dpa^Me^(Pyr^H^)_2_NiI] (right) with thermal ellipsoids at the 50% probability level. One of two crystallographically independent molecules is shown for all complexes. Color code: N, blue; C, gray; Ni, teal; Cl, green; Br; brown; I, purple. Hydrogen atoms are omitted for clarity.

Finally, the persistence of the square planar geometry in pyrrolide analogues, even with relatively weak-field flanking S-donors,^29,30^ highlights the unique electronic potency of the carbazolide unit. The rigid, planar carbazolide backbone can be viewed as an annulated analogue of diphenylamide. Its extended π-conjugation restricts conformational flexibility and enhances the σ- and π-donating ability of the central nitrogen donor, thereby weakening the bond trans to the carbazolide ligand.

This potent trans influence of the carbazolide nitrogen is discernible even when the nickel center is stabilized by strong-field flanking arms (Chart [Fig chart3]). In carbazole-based PCP (phosphine-carbazolide)^31^ or CNC (carbene-carbazolide)^32^ pincer nickel­(II) halide complexes reported in the literature, the metal center generally retains a four-coordinate geometry; however, distinct structural distortions are often observed. Specifically, the Ni–X bond in these species is frequently tilted out of the meridional plane defined by the pincer ligand, or the bond lengths are elongated compared to noncarbazole analogues. While the strong-field phosphine or carbene arms in those systems provide sufficient ligand field stabilization to maintain a diamagnetic state, the large ∠N_Cz_–Ni–X serves as a signature of the electronic repulsion generated by the carbazolide nitrogen.

**3 chart3:**
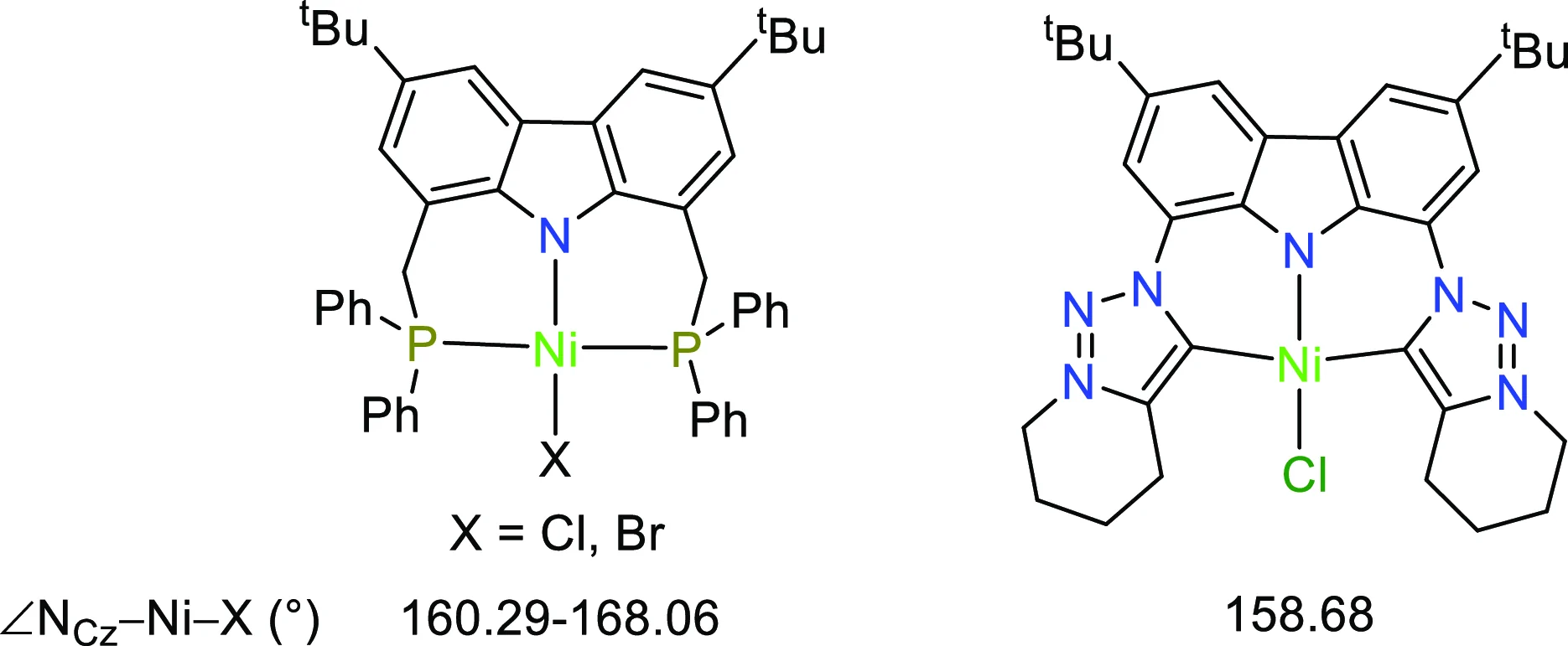
Carbazole-Based PCP and CNC Pincer Nickel­(II) Halide Complexes

## Conclusions

This study demonstrates that carbazolide-based NNN pincer ligands exert a uniquely strong electronic trans influence that significantly alters the coordination chemistry of Ni­(II). Rather than consistently adopting a square-planar geometry, these systems exhibit pronounced coordination plasticity that depends on both the ancillary ligand and the solvent environment. Weakly coordinating anions promote solvation or ionization of the Ni–X bond, oxygen-donor ligands favor dimerization, while strongly donating ligands stabilize square-planar structures.

Comparison with flexible diphenylamide analogues highlights that the rigid, π-conjugated carbazolide backbone, rather than steric effects, is responsible for this behavior. Enhanced σ/π donation from the central nitrogen amplifies the trans influence, labilizing the trans ligand and facilitating access to open coordination sites.

This backbone-driven lability provides a useful design principle for generating reactive Ni­(II) centers and may be advantageous in catalytic processes requiring rapid ligand exchange and substrate activation. Preliminary studies indicate efficient small-molecule binding and C–H activation, and further reactivity investigations are ongoing.

## Experimental Section

All reagents were purchased from chemical vendors and used as received without further purification, unless otherwise specified. No uncommon hazards are noted. All manipulations were performed under a nitrogen atmosphere using standard Schlenk techniques or within a Vigor N_2_-atmosphere glovebox, unless otherwise specified. Glassware was dried at 150 °C overnight. Diethyl ether, *n*-pentane, tetrahydrofuran, and toluene were purified using a solvent purification system (LC Technology Solutions Inc.). Before use, an aliquot of each solvent was tested with a drop of sodium benzophenone ketyl in THF solution. Cz^*t*Bu^(Pyr^*i*Pr^)_2_NiBr and HCz^*t*Bu^(Pyr^H^)_2_ were prepared according to literature procedures. ^3, 18 1^H NMR data were recorded on Bruker 500 MHz spectrometer at 22 °C. Resonances in the ^1^H NMR spectra are referenced to residual C_6_D_5_H at δ = 7.16 ppm, C_4_D_7_HO at δ = 3.58 ppm, CDHCl_2_ at δ = 5.32 ppm, and CHCl_3_ at δ = 7.26 ppm. UV–visible spectra were recorded on a JASCO V-750 spectrophotometer. Mass spectra were recorded on Bruker Autoflex Speed, Triple Quadrupole, MALDI. A more detailed explanation of the experimental and computational details can be found in the Supporting Information.

## Supplementary Material



## Notes

Experimental information and compound characterization data; X-ray crystallographic data; NMR spectra; Cz^*t*Bu^(Pyr^H^)_2_NiCl­(THF)_2_; Cz^*t*Bu^(Pyr^H^)_2_NiF; [Cz^*t*Bu^(Pyr^H^)_2_Ni­(μ-OMe)]_2_; Cz^*t*Bu^(Pyr^H^)_2_NiOBn; [Cz^*t*Bu^(Pyr^H^)_2_Ni­(THF)_3_]­[BAr^F^_4_]; Cz^*t*Bu^(Pyr^H^)_2_NiN­(SiMe_3_)_2_; Cz^*t*Bu^(Pyr^H^)_2_NiCF_3_; Cz^*t*Bu^(Pyr^*i*Pr^)_2_NiF; Dpa^Me^(Pyr^H^)_2_NiCl; Dpa^Me^(Pyr^H^)_2_NiBr; Dpa^Me^(Pyr^H^)_2_NiI (PDF)
