# Supplementary material for: Trans-Influence-Driven Coordination Plasticity in Carbazolide NNN Pincer Nickel(II) Complexes
Source: Inorg Chem. 2026 Aug 22;65(35):20361–8. doi: 10.1021/acs.inorgchem.6c02664 (PMC13556126; doi:10.1021/acs.inorgchem.6c02664)
Supplement: Supplementary file 1 [file ic6c02664_si_001.pdf]

Supplementary Information

Trans-Influence-Driven Coordination Plasticity in Carbazolide NNN Pincer Nickel(II) Complexes

Jun-Yang Ye,[†] *Shu-Chi Liao,*[†] *Matthias Zeller,*[♦] *Wei-Tsung Lee*[*†]

[†] Department of Chemistry, National Central University, Taoyuan 32001, Taiwan.

[♦] Department of Chemistry, Purdue University, West Lafayette, IN 47907, United States.

*Email: wlee5@ncu.edu.tw

Table of Contents

	Page
**General Synthesis of Ni Complexes**	S3
**Conductivity Measurements**	S8
**Crystallography**	S9
**Table S1**. Crystal data and structure refinement detail for $Cz^{tBu}(Pyr^{H})_2NiCl(THF)_2$, $Cz^{tBu}(Pyr^{H})_2NiF$, $[Cz^{tBu}(Pyr^{H})_2Ni(\mu\text{-}OMe)]_2$, and $Cz^{tBu}(Pyr^{H})_2NiOBn$	S10
**Table S2.** Crystal data and structure refinement detail for $[Cz^{tBu}(Pyr^{H})_2Ni(THF)_3][BAr^{F}_4]$, $Cz^{tBu}(Pyr^{H})_2NiN(SiMe_3)_2$, and $Cz^{tBu}(Pyr^{H})_2NiCF_3$	S12
**Table S3.** Crystal data and structure refinement detail for $Dpa^{Me}(Pyr^{H})_2NiCl$, $Dpa^{Me}(Pyr^{H})_2NiBr$, and $Dpa^{Me}(Pyr^{H})_2NiI$	S14
**Figure S1.** ^{1}H NMR spectrum of $Cz^{tBu}(Pyr^{H})_2NiCl$ in $CDCl_3$	S15
**Figure S2.** ^{1}H NMR spectrum of $Cz^{tBu}(Pyr^{H})_2NiBAr^{F}_4$ in $CDCl_3$	S15
**Figure S3.** ^{1}H NMR spectrum of $Cz^{tBu}(Pyr^{H})_2NiN_3$ in $CDCl_3$	S16
**Figure S4.** ^{1}H NMR spectrum of $Cz^{tBu}(Pyr^{H})_2NiF$ in $CDCl_3$ at rt. Inset: ^{19}F NMR spectrum at –40 °C	S16
**Figure S5.** ^{1}H NMR spectrum of $[Cz^{tBu}(Pyr^{H})_2Ni(\mu\text{-}OMe)]_2$ in $CDCl_3$	S17
**Figure S6.** ^{1}H NMR spectrum of $Cz^{tBu}(Pyr^{H})_2NiOBn$ in $CDCl_3$	S17
**Figure S7.** ^{1}H NMR spectrum of $Cz^{tBu}(Pyr^{H})_2NiN(SiMe_3)_2$ in C_6D_6	S18
**Figure S8.** ^{1}H NMR spectrum of $Cz^{tBu}(Pyr^{H})_2NiCF_3$ in $CDCl_3$	S18
**Figure S9.** ^{1}H NMR spectrum of $Dpa^{Me}(Pyr^{H})_2NiCl$ in $CDCl_3$	S19
**Figure S10.** ^{1}H NMR spectrum of $Dpa^{Me}(Pyr^{H})_2NiBr$ in $CDCl_3$	S19
**Figure S11.** ^{1}H NMR spectrum of $Dpa^{Me}(Pyr^{H})_2NiI$ in $CDCl_3$	S20
**Figure S12.** ^{1}H NMR spectroscopic monitoring of the decomposition of $Cz^{tBu}(Pyr^{H})_2NiBr$ in $CDCl_3$	S20
**Figure S13.** Overlay of ^{1}H NMR spectra (500 MHz, in THF-d_8) of $Cz^{tBu}(Pyr^{H})_2NiX$	S21
**Figure S14.** ^{1}H NMR spectrum of $Cz^{tBu}(Pyr^{H})_2NiBr$ in CD_3CN	S21
**Figure S15.** Overlay of ^{1}H NMR spectra of $Cz^{tBu}(Pyr^{H})_2NiF$ in different D-solvent	S22
**Figure S16.** Electronic absorption spectra of $Cz^{tBu}(Pyr^{H})_2NiR$ in THF.	S23
**Figure S17.** Electronic absorption spectra of $[Cz^{tBu}(Pyr^{H})_2Ni(THF)_3]X$ in THF.	S23
**Figure S18**. Molecular structure of $[Cz^{tBu}(Pyr^{iPr})_2Ni(\mu\text{-}F)]_2$.	S24
**Figure S19.** ^{1}H NMR spectrum of $[Cz^{tBu}(Pyr^{iPr})_2Ni(\mu\text{-}F)]_2$ in C_6D_6	S24
**References**	S25

General Synthesis of Ni Complexes

Synthesis of $Cz^{tBu}(Pyr^{H})_2NiCl$. To a Schlenk flask was added 0.124 g (0.301 mmol) of $HCz^{tBu}(Pyr^{H})_2$, 0.032 g (0.301 mmol) of LDA and 0.066g (0.301 mmol) of $NiCl_2(DME)$. The solids were dissolved by adding THF (10 mL), then the mixture was stirred for 48 hrs at 80°C under N_2 atmosphere. After cooling down to room temperature, the solution was filtered through a celite pad, and volatiles were removed under vacuum. The resulting yellow solid was recrystallized from concentrated THF to afford the product as a yellow-green solid (47%). Crystals suitable for X-ray diffraction were grown from a concentrated THF solution at ambient temperature. ^1H NMR (500 MHz, $CDCl_3$, δ): 13.2 (br), 12.4 (br), 9.6, 9.4, 7.9, 3.2, 1.9, 1.6. μ_{eff} ($CDCl_3$): 2.8 ± 0.2 μ_B. UV−vis (THF at 25 °C) λ_{max} (ε [$M^{-1}\cdot cm^{-1}$]): 324.5(25100), 347.5(11300), 404.5(10400), 423.5(12700). HR-MS for $(C_{26}H_{28}N_5Ni)^+$: Calcd, 468.1693; Found, 468.1671. Anal. Calcd for $C_{26}H_{28}ClN_5Ni\cdot(THF)_{0.7}\cdot(H_2O)_{1.6}$: C, 59.23; H, 6.35; N, 11.99. Found: C, 58.97; H, 6.69; N, 12.42.

Synthesis of $[Cz^{tBu}(Pyr^{H})_2Ni(THF)_3]BAr^F{}_4$. 0.100 g (0.182 mmol) of $Cz^{tBu}(Pyr^{H})_2NiBr$ was dissolved in 5 mL of THF and stirred for 5 mins at ambient temperature under N_2 atmosphere. Then 0.178 g (0.182 mmol) of $AgBAr^F{}_4$ in 5 mL THF solution were added, the resulting cloudy solution was stirred for an additional 30 mins at ambient temperature. The solution was filtered through a celite pad and volatiles were removed under vacuum to afford a brown solid. The solid was washed with diethyl ether (10 mL) and dried to yield the product as a brown powder (0.115g, 90%). Crystals suitable for X-ray diffraction were grown from slow evaporation of a chloroform solution at ambient temperature. ^1H NMR (500 MHz, $CDCl_3$, δ): 58.0 (br), 55.2 (br), 41.5 (br), 32.6, 16.2 (br), 10.7, 7.8, 7.6, 5.9 (br), 2.2, 1.5 (br). μ_{eff} ($CDCl_3$): 2.8 ± 0.2 μ_B. UV−vis (THF at 25 °C) λ_{max} (ε [$M^{-1}\cdot cm^{-1}$]): 325(16100), 345.5(8800), 403.5(7500), 423(10100). HR-MS for $(C_{26}H_{28}N_5Ni)^+$: Calcd, 468.1693; Found, 468.1670. Anal. Calcd for $C_{70}H_{64}BF_{24}N_5NiO_3\cdot(AgBr)_{0.5}$: C, 51.18; H, 3.93; N, 4.26. Found: C, 51.52; H, 3.83; N, 4.33.

Synthesis of $Cz^{tBu}(Pyr^{H})_2NiN_3$. 0.100 g (0.182 mmol) of $Cz^{tBu}(Pyr^{H})_2NiBr$ was dissolved in 5 mL of THF and stirred for 5 mins at ambient temperature under N_2 atmosphere. Then 0.024 g (0.728 mmol) of NaN_3 in 5 mL THF solution were added, the mixture was stirred for 8 hrs at ambient temperature. The solution was filtered through a celite pad and volatiles were removed under vacuum to afford a dark red solid. The solid was washed with *n*-pentane (3 x 10 mL) and dried to yield the product as a dark red powder (0.082g, 87%). ^{1}H NMR (500 MHz, $CDCl_3$, δ): 8.70 (br, 2H, ArH), 8.18 (br, 4H, ArH), 7.50 (br, 2H, ArH), 6.81 (br, 2H, ArH), 1.50 (s, 18H, $C(CH_3)_3$). UV−vis (THF at 25 °C) λ_{max} (ε [$M^{-1}\cdot cm^{-1}$]): 324.5(20200), 345.5(10400), 403.5(8200), 424.5(11100).

Synthesis of $Cz^{tBu}(Pyr^{H})_2NiF$. 0.050 g (0.091 mmol) of $Cz^{tBu}(Pyr^{H})_2NiBr$ was dissolved in 5 mL of THF and stirred for 5 mins at ambient temperature under N_2 atmosphere. Then 0.041 g (0.273 mmol) of CsF in 10 mL THF solution were added, the mixture was stirred for 48 hrs at ambient temperature. The solution was filtered through a celite pad and volatiles were removed under vacuum to afford a green solid. The solid was washed with toluene (5 mL) and dried to yield the product as a bright green powder (0.021g, 45%). Crystals suitable for X-ray diffraction were grown from slow evaporation of a chloroform solution at ambient temperature. ^{1}H NMR (500 MHz, $CDCl_3$, δ): 8.45 (s, 2H, ArH), 7.96 (s, 2H, ArH), 7.90 (s, 2H, ArH), 7.48 (s, 2H, ArH), 6.61 (s, 2H, ArH), 1.47 (s, 18H, $C(CH_3)_3$). UV−vis (THF at 25 °C) λ_{max} (ε [$M^{-1}\cdot cm^{-1}$]): 323.5(159000), 340.5(8400), 354(8000), 407(7800), 430(12100). Anal. Calcd for $C_{26}H_{28}FN_5Ni$: C, 63.96; H, 5.78; N, 14.34. Found: C, 64.01; H, 5.93; N, 13.96.

Synthesis of $[Cz^{tBu}(Pyr^{H})_2Ni(\mu\text{-}OMe)]_2$. To a vial was added 0.030 g (0.055 mmol) of $Cz^{tBu}(Pyr^{H})_2NiBr$ and 0.006 g (0.110 mmol) of NaOMe. The solids were dissolved by adding THF (10 mL), then the mixture was stirred for 30 mins at ambient temperature under N_2 atmosphere. The resulting cloudy solution was filtered through a celite pad and volatiles were removed under vacuum to afford a yellow solid. The solid was washed with *n*-pentane (5 mL)

and dried to yield the product as a yellow-green powder (0.020g, 40%). Crystals suitable for X-ray diffraction were grown by slow diffusion *n*-pentane into DCM solution at −30 °C. ^{1}H NMR (500 MHz, $CDCl_3$, δ): 8.36 (br, 2H, ArH), 7.96 (br, 4H, ArH), 7.47 (br, 2H, ArH), 6.58 (br, 2H, ArH), 3.43 (br, 3H, OCH_3), 1.50 (s, 18H, $C(CH_3)_3$). UV−vis (THF at 25 °C) λ_{max} (ε [$M^{-1}\cdot cm^{-1}$]): 324(15691), 339(8300), 353(8100), 408.5(7100), 430(11500), 488.5(200). Anal. Calcd for $C_{54}H_{62}N_{10}Ni_2O_2$: C, 64.82; H, 6.25; N, 14.00. Found: C, 63,76; H, 5.99; N, 13.65.

Synthesis of $Cz^{tBu}(Pyr^{H})_2NiOBn$. 0.050 g (0.091 mmol) of $Cz^{tBu}(Pyr^{H})_2NiOMe$ was dissolved in 5 mL of THF and stirred for 5 mins at ambient temperature under N_2 atmosphere. Then 0.011 g (0.100 mmol) of BnOH in 5 mL THF solution were added, the mixture was stirred for an additional 5 mins at ambient temperature. Volatiles were removed under vacuum to afford a dark green solid, the solid was washed with *n*-pentane (5 mL) and dried to yield the product as a dark-green powder (0.048g, 83%). Crystals suitable for X-ray diffraction were grown from slow evaporation of a *n*-pentane solution at ambient temperature. ^{1}H NMR (500 MHz, $CDCl_3$, δ): 8.40 (br, 4H, ArH), 8.01 (br, 2H, ArH), 7.49 (br, 2H, ArH), 7.38 (br, 2H, ArH), 7.29 (br, 2H, ArH), 7.20 (br, 1H, ArH), 6.57 (br, 2H, ArH), 4.73 (br, 2H, CH_2), 1.49 (s, 18H, $C(CH_3)_3$). UV−vis (THF at 25 °C) λ_{max} (ε [$M^{-1}\cdot cm^{-1}$]): 324(17800), 340(9400), 353.5(9500), 408.5(8200), 430.5(13500), 488(300). Anal. Calcd for $C_{33}H_{35}N_5NiO$: C, 68.77; H, 6.12; N, 12.15. Found: C, 67.55; H, 6.17; N, 11.75.

Synthesis of $Cz^{tBu}(Pyr^{H})_2NiN(SiMe_3)_2$. 0.200 g (0.364 mmol) of $Cz^{tBu}(Pyr^{H})_2NiBr$ was dissolved in 10 mL of THF and stirred for 5 mins at ambient temperature under N_2 atmosphere. Then 0.067 g (0.400 mmol) of $LiN(SiMe_3)_2$ in 5 mL THF solution were added, the mixture was stirred for an additional 30 mins at ambient temperature. Volatiles were removed under vacuum, the resulting brown solid was extracted with *n*-pentane (10 mL) and filtered through a celite pad. The filtrate was dried *in vacuo* to afford the product as a green powder (0.176g, 77%). Crystals suitable for X-ray diffraction were grown from slow evaporation of *n*-pentane

at ambient temperature. ^{1}H NMR (500 MHz, C_6D_6, δ): 8.73 (s, 2H, ArH), 8.12 (s, 2H, ArH), 7.41 (s, 2H, ArH), 7.08 (s, 2H, ArH), 5.84 (s, 2H, ArH), 1.41 (s, 18H, $C(CH_3)_3$), 0.86 (s, 18H, $Si(CH_3)_3$). UV−vis (THF at 25 °C) λ_{max} (ε [$M^{-1}\cdot cm^{-1}$]): 324(16200), 345(8200), 360.5(6300), 412(8100), 432.5(11200). Anal. Calcd for $C_{32}H_{46}N_6NiSi_2\cdot(LiBr)_{0.5}$: C, 57.11; H, 6.89; N, 12.49. Found: C, 57.31; H, 6.42 N, 12.28.

Synthesis of $Cz^{tBu}(Pyr^H)_2NiCF_3$. 0.050 g (0.091 mmol) of $Cz^{tBu}(Pyr^H)_2NiF$ was dissolved in 5 mL of THF and stirred for 5 mins at ambient temperature under N_2 atmosphere. Then 0.015 g (0.091 mmol) of $TMSCF_3$ in 5 mL THF solution were added, the mixture was stirred for an additional 5 mins at ambient temperature. The solution was concentrated to *ca.* 2 mL by vacuum, then 10 mL *n*-pentane solution was added to result in an orange suspension. The suspension was filtered through a celite pad and washed with *n*-pentane (2 x 5 mL), the remaining solid was dissolved with toluene, volatiles were removed under vacuum to afford the final product as an orange powder. Crystals suitable for X-ray diffraction were grown by slow diffusion of *n*-pentane into toluene solution at −30 °C. ^{1}H NMR (500 MHz, $CDCl_3$, δ): 8.25 (s, 4H, ArH), 8.09 (s, 2H, ArH), 7.44 (s, 2H, ArH), 6.62 (s, 2H, ArH), 1.50 (s, 18H, $C(CH_3)_3$). UV−vis (THF at 25 °C) λ_{max} (ε [$M^{-1}\cdot cm^{-1}$]): 323(16000), 344.5(8200), 403.5(8200), 421.5(11500), 528.5(400). Anal. Calcd for $C_{27}H_{28}F_3N_5Ni$: C, 60.25; H, 5.24; N, 13.01. Found: C, 59.89; H, 5.25; N, 12.88.

Synthesis of *$DPA^{Me}(Pyr^H)_2NiCl$.* To a solution of $DPA^{Me}(Pyr^H)_2$ (0.072 g, 0.219 mmol, 1.0 equiv) in THF (2 mL) in a Schlenk tube at ambient temperature under a N_2 atmosphere were added a solution of KO^{t}Bu (0.027 g, 0.241 mmol, 1.1 equiv) in THF (1 mL). The reaction mixture was stirred for 5 min, then $NiCl_2$(DME) (0.053 g, 0.241 mmol, 1.1 equiv) in THF (3 mL) were added. The mixture was heated at 80 °C for 16 h. Upon completion of the reaction, volatiles were removed under reduced pressure, and the crude product was recrystallized from a THF/toluene solution to afford an olive solid (0.078 g, 84%). Crystals suitable for X-ray

diffraction were grown by slow diffusion of *n*-pentane into a toluene solution at ambient temperature. ^{1}H NMR (500 MHz, $CDCl_3$, δ): 8.02 (s, 2H, ArH), 7.87 (s, 2H, ArH), 7.08 (s, 2H, ArH), 6.83 (s, 4H, ArH), 6.43 (s, 2H, ArH), 2.31 (s, 6H, CH_3). UV−vis (toluene at 25 °C) λ_{max} (ε [$M^{-1}\cdot cm^{-1}$]): 341.5 (12000), 408 (8500), 517.5 (200), 693 (500). HR-MS for $(C_{20}H_{18}ClN_5Ni)^+$: Calcd, 421.0604; Found, 421.0615. Anal. Calcd for $C_{20}H_{18}ClN_5Ni$: C, 56.85; H, 4.29; N, 16.57. Found: C, 56.43; H, 4.46; N, 15.74.

Synthesis of *$DPA^{Me}(Pyr^{H})_2NiBr$.* To a solution of $DPA^{Me}(Pyr^{H})_2$ (0.146 g, 0.443 mmol, 1.0 equiv) in THF (3 mL) in a Schlenk tube at ambient temperature under a N_2 atmosphere were added a solution of KO*t*Bu (0.055 g, 0.488 mmol, 1.1 equiv) in THF (2 mL). The reaction mixture was stirred for 5 min, then $NiBr_2(THF)_{1.5}$ (0.150 g, 0.488 mmol, 1.1 equiv) in THF (3 mL) was added. The mixture was stirred overnight. Upon completion of the reaction, volatiles were removed under reduced pressure, and the crude product was recrystallized from a THF/toluene solution to afford an orange-brown solid (0.184 g, 89%). Crystals suitable for X-ray diffraction were grown by slow diffusion of *n*-pentane into a toluene solution at ambient temperature. ^{1}H NMR (500 MHz, $CDCl_3$, δ): 8.20 (s, 2H, ArH), 7.85 (s, 2H, ArH), 7.07 (s, 2H, ArH), 6.83 (s, 4H, ArH), 6.42 (s, 2H, ArH), 2.31 (s, 6H, CH_3). UV−vis (toluene at 25 °C) λ_{max} (ε [$M^{-1}\cdot cm^{-1}$]): 340.5 (7600), 408.5 (4800), 517 (200), 717 (300). HR-MS for $(C_{20}H_{18}BrN_5Ni)^+$: Calcd, 465.0099; Found, 465.0114. Anal. Calcd for $C_{20}H_{18}BrN_5Ni$: C, 51.44; H, 3.89; N, 15.00. Found: C, 51.07; H, 3.92; N, 14.44.

Synthesis of *$DPA^{Me}(Pyr^{H})_2NiI$.* To a solution of $DPA^{Me}(Pyr^{H})_2$ (0.070 g, 0.213 mmol, 1.0 equiv) in THF (2 mL) in a Schlenk tube at ambient temperature under a N_2 atmosphere were added a solution of KO*t*Bu (0.026 g, 0.234 mmol, 1.1 equiv) in THF (1 mL). The reaction mixture was stirred for 5 min, then NiI_2 (0.073 g, 0.234 mmol, 1.1 equiv) in THF (3 mL) was added. The mixture was heated at 80 °C for 24 h. Upon completion of the reaction, volatiles were removed under reduced pressure, and the crude product was recrystallized from a THF/toluene solution

to afford an olive solid (0.078 g, 84%). Crystals suitable for X-ray diffraction were grown by slow diffusion of *n*-pentane into a THF solution at ambient temperature. ^{1}H NMR (500 MHz, $CDCl_3$, δ): 8.45 (s, 2H, ArH), 7.84 (s, 2H, ArH), 6.83 (s, 2H, ArH), 6.82 (s, 4H, ArH), 6.39 (s, 2H, ArH), 2.32 (s, 6H, CH_3). UV−vis(toluene at 25 °C) λ_{max} (ε [$M^{-1}\cdot cm^{-1}$]): 374(4600), 389.5(5100), 528.5(2100). HR-MS for $(C_{20}H_{18}IN_5Ni)^+$: Calcd, 512.9955; Found, 512.9979. Anal. Calcd for $C_{20}H_{18}IN_5Ni$: C, 46.74; H, 3.53; N, 13.63. Found: C, 46.04; H, 3.47; N, 13.31.

Synthesis of $[Cz^{tBu}(Pyr^{iPr})_2Ni(\mu\text{-}F)]_2$. 0.063 g (0.100 mmol) of $Cz^{tBu}(Pyr^{iPr})_2NiBr$ was dissolved in 10 mL of THF and stirred for 5 mins at ambient temperature under N_2 atmosphere. Then 0.076 g (0.500 mmol) of CsF solid was added, the suspension was stirred for 12 h at ambient temperature. The cloudy orange solution was filtered through a celite pad and volatiles were removed under vacuum. The resulting dark orange oil was recrystallized from THF/*n*-pentane solution at ambient temperature to afford the final product as a bright orange powder (0.034 g, 60%). Crystals suitable for X-ray diffraction were grown by slow diffusion of *n*-pentane into THF solution at ambient temperature. ^{1}H NMR (500 MHz, C_6D_6, δ): 41.7, 29.9, 26.3 (br), 24.8, 12.7, 6.3, 4.9, –6.5. μ_{eff} (C_6D_6): 2.8 ± 0.2 μ_B. Anal. Calcd for $C_{64}H_{80}F_2N_{10}Ni_2$: C, 67.15; H, 7.04; N, 12.24. Found: C, 66.63; H, 6.99; N, 12.03.

Conductivity Measurements

The ionic conductivity (σ) was determined via electrochemical impedance spectroscopy (EIS) using a CHI 6114E analyzer. A custom conductivity cell with two parallel high-purity graphite rods was employed. The cell constant (K) was calibrated using a standard 1408 μS/cm KCl aqueous solution at 25 °C following the OIML R56 recommendation.[1] EIS was performed at open-circuit potential (OCP) from 100 kHz to 0.1 Hz. The large amplitude was utilized for high-impedance samples to enhance the signal-to-noise ratio. The solution resistance (R_s) was extracted from the Nyquist plot at the point where the capacitive loop transitions to the

diffusion-controlled regime (Warburg impedance).[2] The conductivity was calculated using the equation: $\sigma = K/R_s$. All measurements were conducted at a controlled temperature of 25 °C.[3]

Crystallography

Data were collected using a Bruker Quest CMOS diffractometer with a sealed tube X-ray source with graphite monochromated Mo-Kα radiation (λ = 0.71073 Å). A single crystal of each compound was mounted on a Mitegen micromesh mount using a trace of Fomblin oil and cooled *in situ* to 100 or 150 K for data collection. Frames were collected, reflections were indexed and processed, and the files scaled and corrected for absorption using Apex3 or Apex5.[4] For all structures, the intensity data were corrected for absorption using multi-scan techniques (SADABS or TWINABS).[5] The space groups were assigned and the structures were solved by direct or dual-space methods using XPREP within the SHELXTL suite of programs and refined by full matrix least squares against F^2 with all reflections using Shelxl 2016 or 2019 using the graphical interface Shelxle.[6-8] If not specified otherwise, H atoms attached to carbon and nitrogen atoms and hydroxyl hydrogens were positioned geometrically and constrained to ride on their parent atoms, with carbon hydrogen bond distances of 0.95 Å for alkene and aromatic C−H, and 0.99 and 0.98 Å for aliphatic CH_2 and CH_3 moieties, respectively. Methyl H atoms were allowed to rotate but not to tip to best fit the experimental electron density. $U_{iso}(H)$ values were set to a multiple of $U_{eq}(C)$ with 1.5 for CH_3 and 1.2 for CH_2 and CH units, respectively. Additional experimental details for all structures are given in the SI. Structures, in CIF format, have been deposited with the Cambridge Crystallographic Data Centre, CCDC 2556508-2556518. These data can be obtained free of charge from The Cambridge Crystallographic Data Centre via www.ccdc.cam.ac.uk/data_request/cif.

Table S1. Crystal data and structure refinement detail for $Cz^{tBu}(Pyr^{H})_2NiCl(THF)_2$, $Cz^{tBu}(Pyr^{H})_2NiF$, $[Cz^{tBu}(Pyr^{H})_2Ni(\mu\text{-}OMe)]_2$, and $Cz^{tBu}(Pyr^{H})_2NiOBn$.

	$Cz^{tBu}(Pyr^{H})_2NiCl(THF)_2$ [a]	$Cz^{tBu}(Pyr^{H})_2NiF$	$[Cz^{tBu}(Pyr^{H})_2Ni(\mu\text{-}OMe)]_2$ [b]	$Cz^{tBu}(Pyr^{H})_2NiOBn$
Empirical formula	$C_{34}H_{44}ClN_5NiO_2$	$C_{26}H_{28}FN_5Ni$, $(CHCl_3)$	$3(C_{54}H_{62}N_{10}Ni_2O_2)$, $8(CH_2Cl_2)$	$C_{33}H_{35}N_5NiO$
Formula weight	648.88	607.59	3680.96	576.35
Space group	$P2_1/c$	$P2_1/c$	$C2/c$	$P2_1/n$
a/Å	25.637(3)	15.793(11)	37.55(3)	11.5391(5)
b/Å	13.9266(15)	5.837(3)	12.502(7)	14.9305(5)
c/Å	19.3085(18)	29.98(2)	42.64(4)	16.4684(7)
a/°	90	90	90	90
b/°	111.726(4)	91.38(2)	94.22(3)	96.2410(10)
g/°	90	90	90	90
V/Å^3	6404.2(12)	2763(3)	19961(26)	2820.4(2)
Z	8	4	4	4
D_{calcd}, g cm^{-3}	1.346	1.461	1.225	1.357
F(000)	2752	1256	7680	1216
Temp, K	150	100	150	150
R(F), %	8.95	7.14	8.97	6.83
R_w(F), %	15.61	20.01	21.55	12.18

Special refinement details:

[a] The crystal of $Cz^{tBu}(Pyr^{H})_2NiCl(THF)_2$ under investigation was found to be twinned by emulating a double volume orthorhombic C-centered lattice and was refined as a 2-component twin. Twin matrix 1 0 1 0 -1 0 0 0 -1. The twin ratio refined to 0.708(5) to 0.292(5). One THF molecule was refined as disordered. The major and minor disordered moieties were each

restrained to have similar geometries. U^{ij} components of ADPs for disordered atoms closer to each other than 2.0 Angstrom were restrained to be similar. Subject to these conditions the occupancy ratio refined to 0.38(2) to 0.62(2).

[b] In the structure of $[Cz^{tBu}(Pyr^{H})_2Ni(\mu\text{-}OMe)]_2$ five tert-butyl groups were refined as disordered by rotation. The major and minor moieties were each restrained to have similar geometries. U^{ij} components of ADPs for disordered atoms closer to each other than 2.0 Angstrom were restrained to be similar. Subject to these conditions the occupancy ratio refined to 0.54(5) to 0.46(5) for ^{t}Bu of C19, 0.587(9) to 0.413(9) for ^{t}Bu of C46, 0.692(9) to 0.308(9) for ^{t}Bu of C50, 0.70(3) to 0.30(3) for ^{t}Bu of C73, 0.477(9) to 0.523(9) for ^{t}Bu of C77. Three solvate methylene chloride molecules were refined as disordered in different orientation. All moieties were restrained to have similar geometries with non-disordered molecule(C86). U^{ij} components of ADPs for disordered atoms closer to each other than 2.0 Angstrom were restrained to be similar. Subject to these conditions the occupancy ratio refined to 0.673(11) to 0.327(11) for molecule of C82, 0.553(11) to 0.447(11) for molecule of C83, 0.164(6) to 0.836(6) for molecule of C84. The structure contains additional 2406 Ang3 of solvent accessible pores. No substantial electron density peaks were found in the solvent accessible pores and the residual electron density peaks are not arranged in an interpretable pattern. The structure factors were instead augmented via reverse Fourier transform methods using the SQUEEZE routine (P. van der Sluis & A.L. Spek (1990). Acta Cryst. A46, 194-201) as implemented in the program Platon. The resultant FAB file containing the structure factor contribution from the electron content of the pore space was used in together with the original hkl file in the further refinement. (The FAB file with details of the Squeeze results is appended to this cif file). The Squeeze procedure corrected for 806 electrons within the solvent accessible pores.

Table S2. Crystal data and structure refinement detail for $[Cz^{tBu}(Pyr^{H})_2Ni(THF)_3][BAr^{F}_4]$, $Cz^{tBu}(Pyr^{H})_2NiN(SiMe_3)_2$, $Cz^{tBu}(Pyr^{H})_2NiCF_3$ and $[Cz^{tBu}(Pyr^{iPr})_2Ni(\mu\text{-}F)]_2$.

	[Cz^{tBu}(Pyr^{H})$_2$Ni(THF)$_3$][BAr$^{F}_4$][c]	*Cz^{tBu}(Pyr^{H})$_2$NiN(SiMe$_3$)$_2$*	*Cz^{tBu}(Pyr^{H})$_2$NiCF$_3$*[d]	*[Cz^{tBu}(Pyr^{iPr})$_2$Ni(μ-F)]$_2$*[e]
Empirical formula	$(C_{38}H_{52}N_5NiO_3)$, $(C_{32}H_{12}BF_{24})$, $2.5(CHCl_3)$	$C_{32}H_{46}N_6NiSi_2$	$2(C_{27}H_{28}FN_5Ni)$, (C_5H_{12})	$C_{64}H_{80}F_2N_{10}Ni_2$, $6(C_4H_8O)$
Formula weight	1847.06	629.62	1148.61	1577.42
Space group	$P2_1/n$	$Fdd2$	$C2/c$	$P2_1/n$
a/Å	17.4467(11)	21.5475(10)	21.4052(11)	13.3447(5)
b/Å	26.6201(16)	16.9853(7)	15.4454(7)	16.3720(9)
c/Å	17.8456(10)	18.5777(7)	17.5302(10)	19.9239(12)
a/°	90	90	90	90
b/°	95.272(3)	90	107.584(2)	104.348(2)
g/°	90	90	90	90
V/Å^3	8253.0(9)	6799.3(5)	5524.9(5)	4217.2(4)
Z	4	8	4	2
D_{calcd}, g cm^{-3}	1.487	1.230	1.381	1.242
F(000)	3748	2688	2408	1696
Temp, K	150	150	150	100
R(F), %	9.38	4.63	5.61	4.57
R_w(F), %	22.82	7.98	13.04	8.68

Special refinement details:

[c] In the structure of $[Cz^{tBu}(Pyr^{H})_2Ni(THF)_3][BAr^{F}_4]$ three chloroform molecules were refined as disordered. One of the molecules (that of C71) is refined as rotationally disordered, the molecule of C73 is refined as disordered around an inversion center and the remaining molecule is refined as three-fold disordered in different orientations (molecule of C72). The major and minor disordered moieties are each restrained to have similar geometries. U^{ij}

components of ADPs for disordered atoms closer to each other than 2.0 Angstrom were restrained to be similar. Subject to these conditions the occupancy ratio refined to 0.552(11) to 0.448(11) (C71), 0.438(3) to 0.374(3) to 0.188(3) (C72). Seven trifluoromethyl groups were refined as disordered by rotation. The major and minor moieties were each restrained to have similar geometries. U^{ij} components of ADPs for disordered atoms closer to each other than 2.0 Angstrom were restrained to be similar. Subject to these conditions the occupancy ratio refined to 0.625(15) to 0.375(15) (C45), 0.65(2) to 0.35(2) (C53), 0.548(7) to 0.452(7) (C54), 0.790(7) to 0.210(7) (C61), 0.814(12) to 0.186(7) (C62), 0.283(10) to 0.717(10) (C69), 0.72(2) to 0.28(2) (C70).

[d] In the structure of $Cz^{tBu}(Pyr^{H})_2NiCF_3$ one tert-butyl group was refined as disordered by rotation. The major and minor moieties were each restrained to have similar geometries using SAME restraints. U^{ij} components of ADPs for disordered atoms closer to each other than 2.0 Angstrom were restrained to be similar. Subject to these conditions the occupancy ratio refined to 0.54(3) to 0.46(3).

[e] In the structure of $[Cz^{tBu}(Pyr^{iPr})_2Ni(\mu\text{-}F)]_2$ a tert-butyl, and isopropyl and three solvate THF molecules were refined as disordered over each two orientations. Major and minor moieties were each restrained to have similar geometries, and U^{ij} components of ADPs were restrained to be similar for atoms closer to each other than 1.7 Angstrom. The occupancy ratios of two of the THF molecules are coupled to that of the tert-butyl group. Subject to these conditions the occupancy ratios refined to 0.664(3) to 0.336(3) (tert-butyl), 0.790(7) to 0.210(7) (isopropyl), and 0.541(15) to 0.459(15) (third THF).

Table S3. Crystal data and structure refinement detail for $Dpa^{Me}(Pyr^{H})_2NiCl$, $Dpa^{Me}(Pyr^{H})_2NiBr$, and $Dpa^{Me}(Pyr^{H})_2NiI$.

	$Dpa^{Me}(Pyr^{H})_2NiCl$	***$Dpa^{Me}(Pyr^{H})_2NiBr$***	***$Dpa^{Me}(Pyr^{H})_2NiI$***
Empirical formula	$C_{20}H_{18}ClN_5Ni$	$C_{20}H_{18}BrN_5Ni$	$C_{40}H_{36}I_{1.56}N_{10}Ni_2$
Formula weight	422.53	466.98	972.00
Space group	$P2_12_12_1$	*Pnna*	*Pnna*
a/Å	10.7264(8)	9.7616(4)	9.7337(13)
b/Å	13.6064(8)	26.7560(14)	26.660(4)
c/Å	25.7087(18)	14.3621(7)	14.3519(18)
a/°	90	90	90
b/°	90	90	90
g/°	90	90	90
V/Å^3	3752.1(4)	3751.1(3)	3724.3(9)
Z	8	8	4
D_{calcd}, g cm^{-3}	1.496	1.654	1.733
F(000)	1744	1888	1938
Temp, K	150	150	150
R(F), %	4.11	3.01	3.65
R_w(F), %	7.98	7.27	7.70

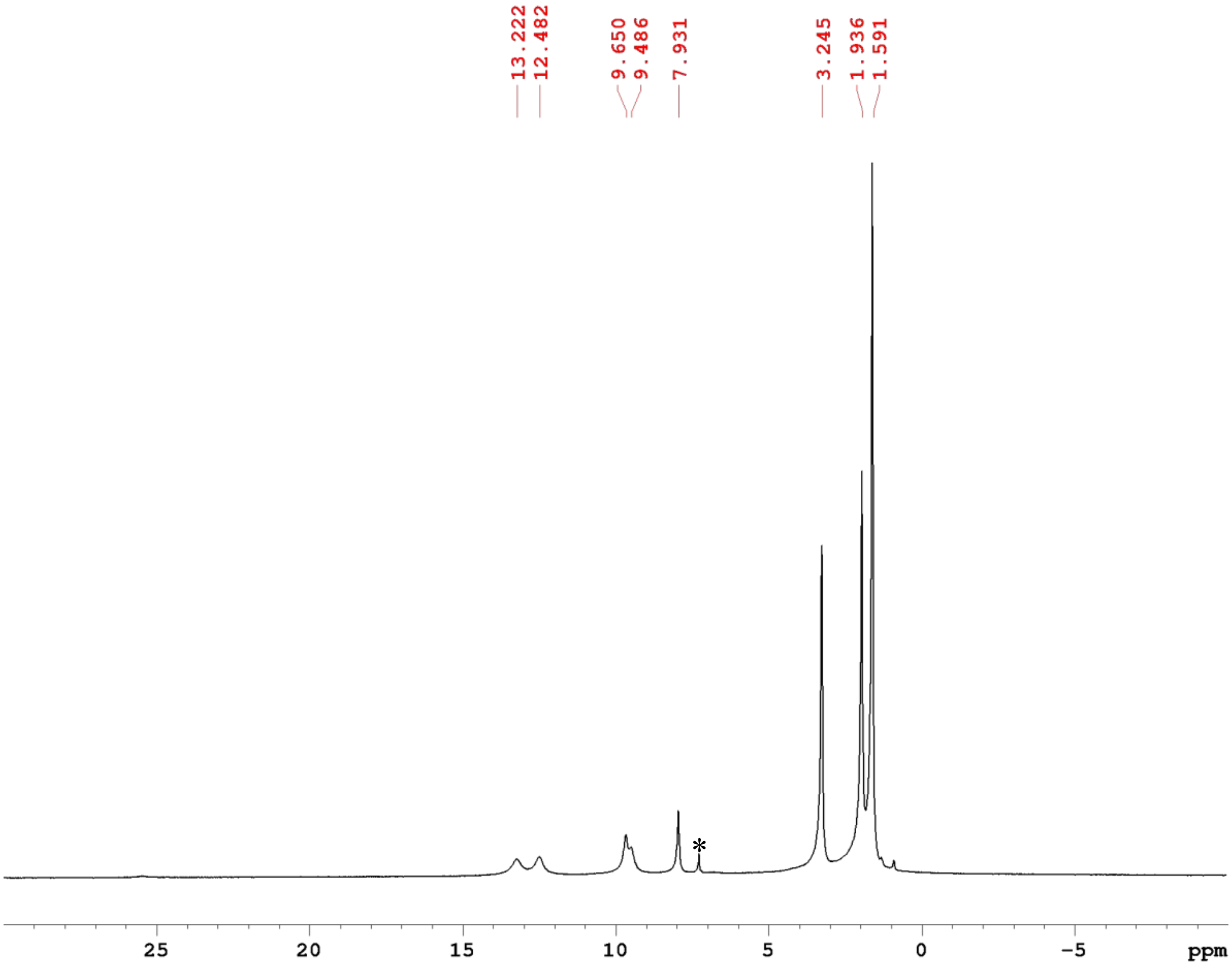

Figure S1. ^{1}H NMR spectrum of $Cz^{tBu}(Pyr^H)_2NiCl$ in $CDCl_3$. * solvent residual peaks.

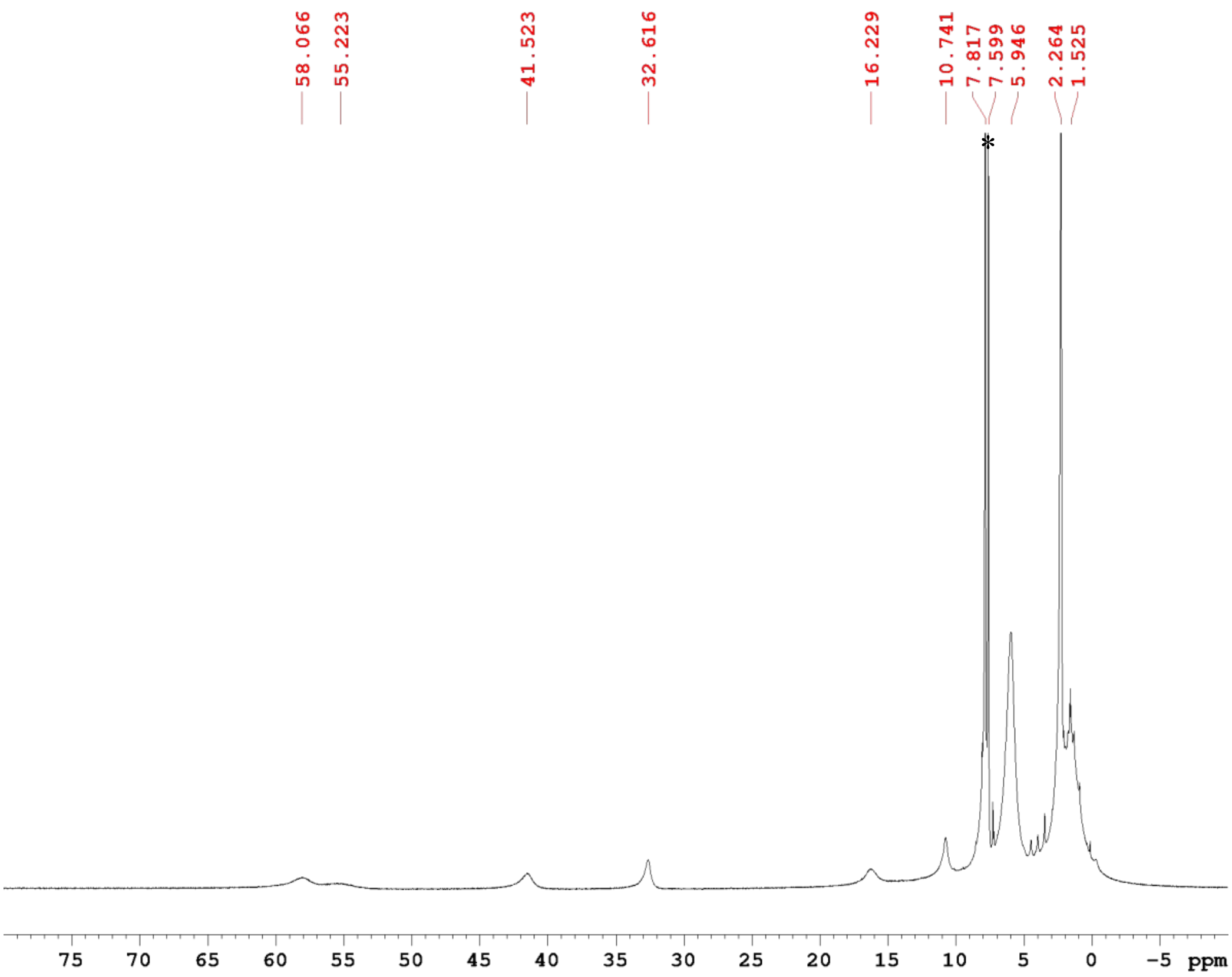

Figure S2. ^{1}H NMR spectrum of $Cz^{tBu}(Pyr^H)_2NiBAr^F{}_4$ in $CDCl_3$. * solvent residual peaks.

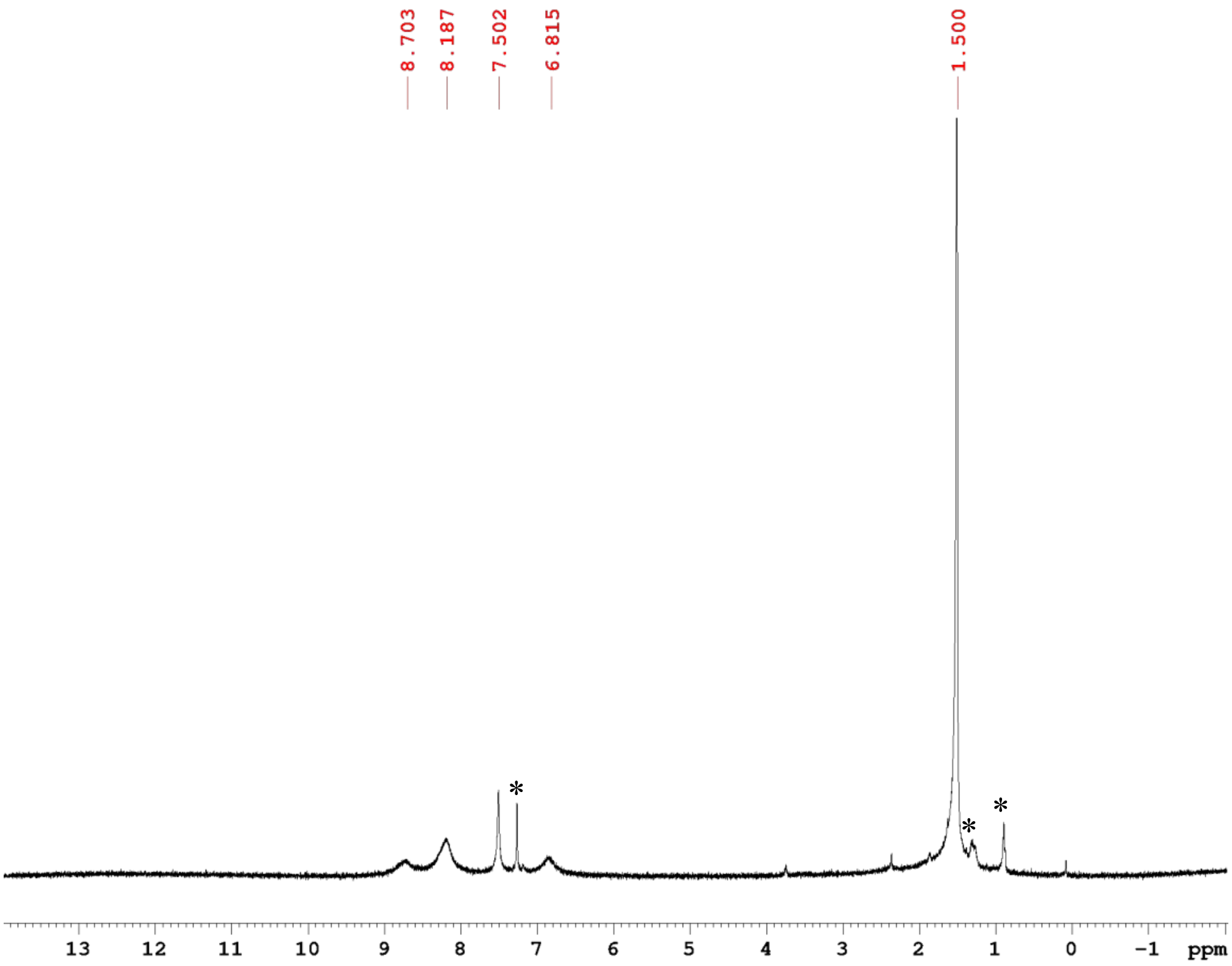

Figure S3. ^{1}H NMR spectrum of $Cz^{tBu}(Pyr^{H})_2NiN_3$ in $CDCl_3$. * solvent residual peaks.

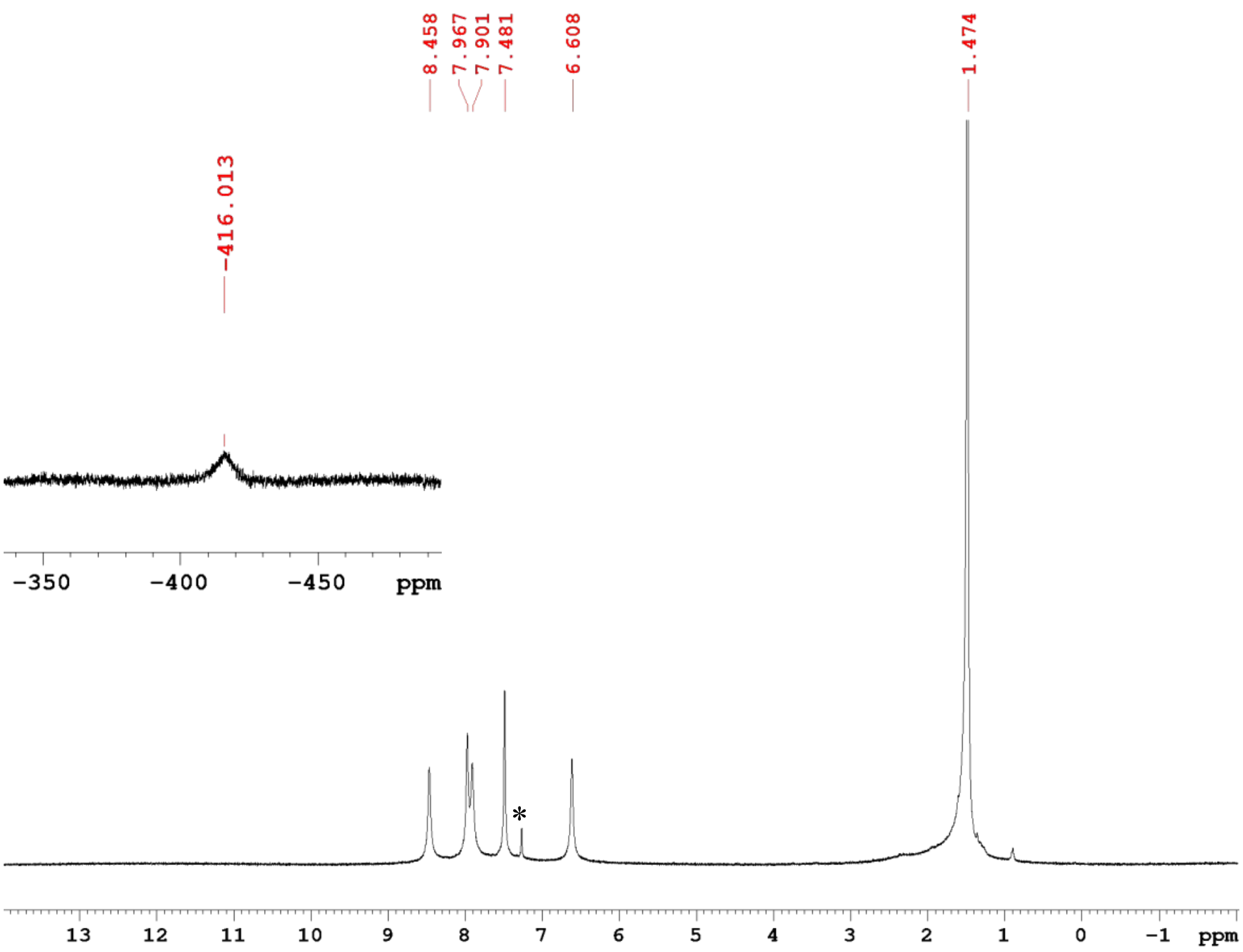

Figure S4. ^{1}H NMR spectrum of $Cz^{tBu}(Pyr^{H})_2NiF$ in $CDCl_3$. Inset: ^{19}F NMR spectrum at -40°C. * solvent residual peaks.

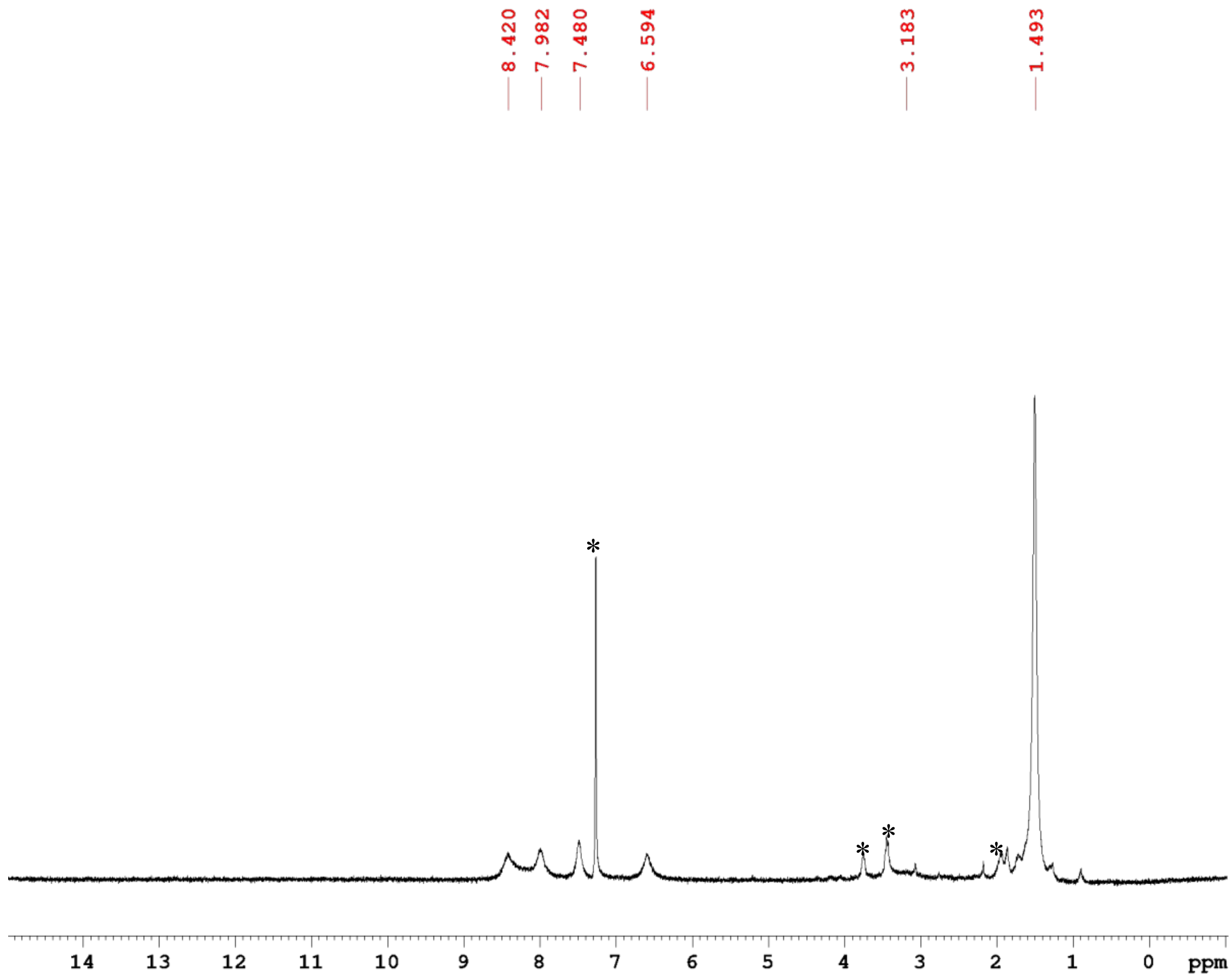

Figure S5. ^{1}H NMR spectrum of $[Cz^{tBu}(Pyr^{H})_2Ni(\mu\text{-}OMe)]_2$ in $CDCl_3$. * solvent residual peaks.

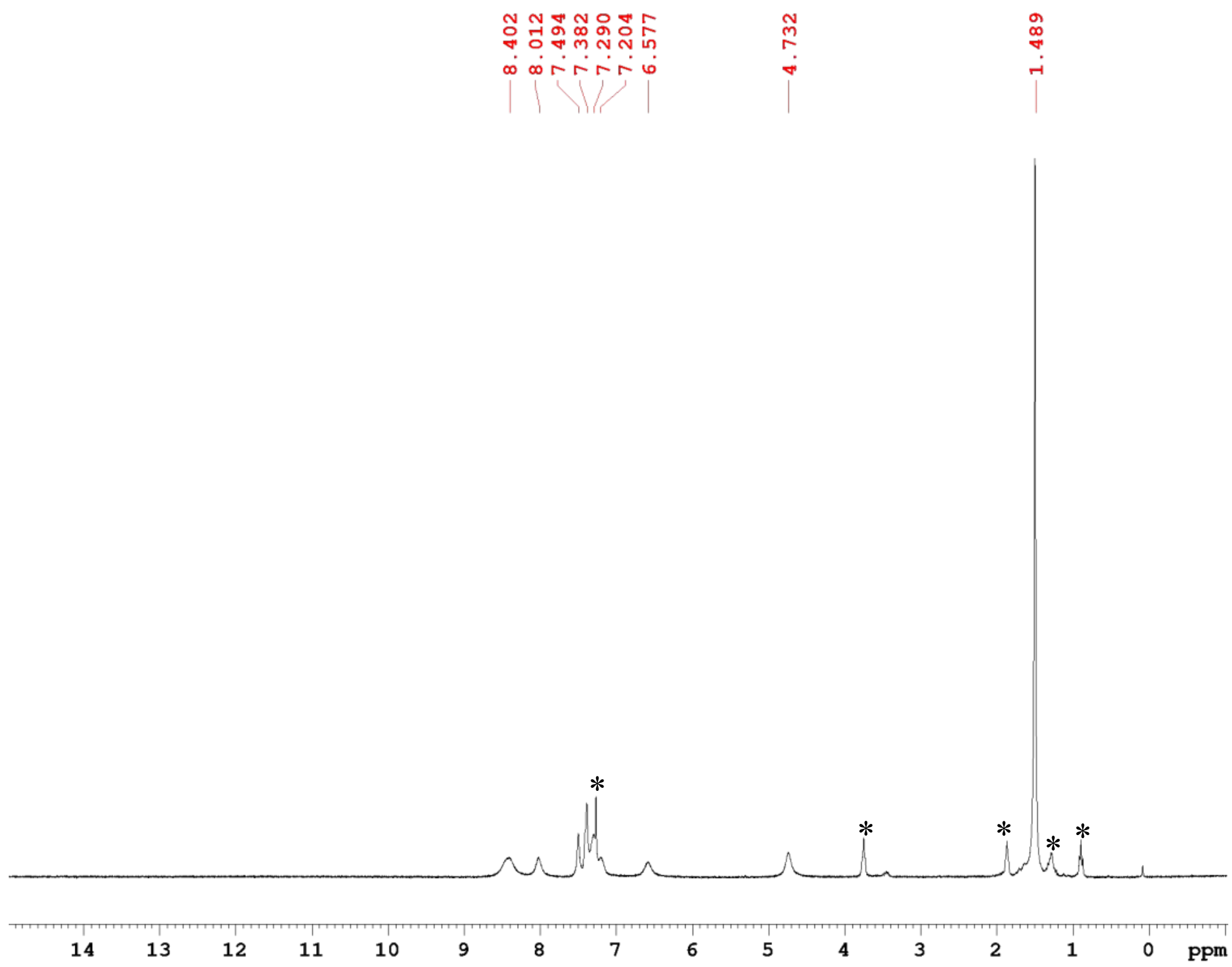

Figure S6. ^{1}H NMR spectrum of $Cz^{tBu}(Pyr^{H})_2NiOBn$ in $CDCl_3$. * solvent residual peaks.

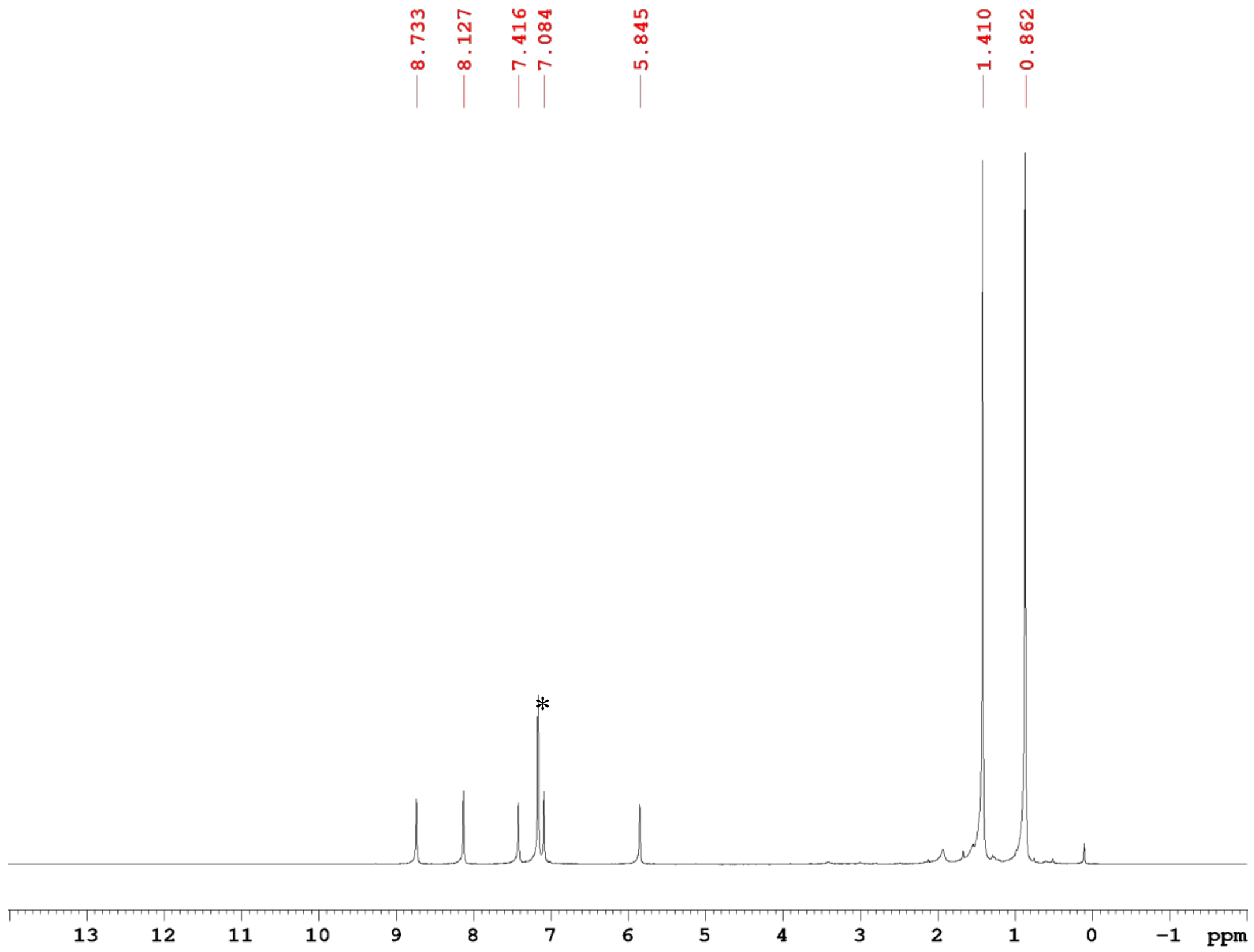

Figure S7. ^{1}H NMR spectrum of $Cz^{tBu}(Pyr^{H})_2NiN(SiMe_3)_2$ in C_6D_6. * solvent residual peaks.

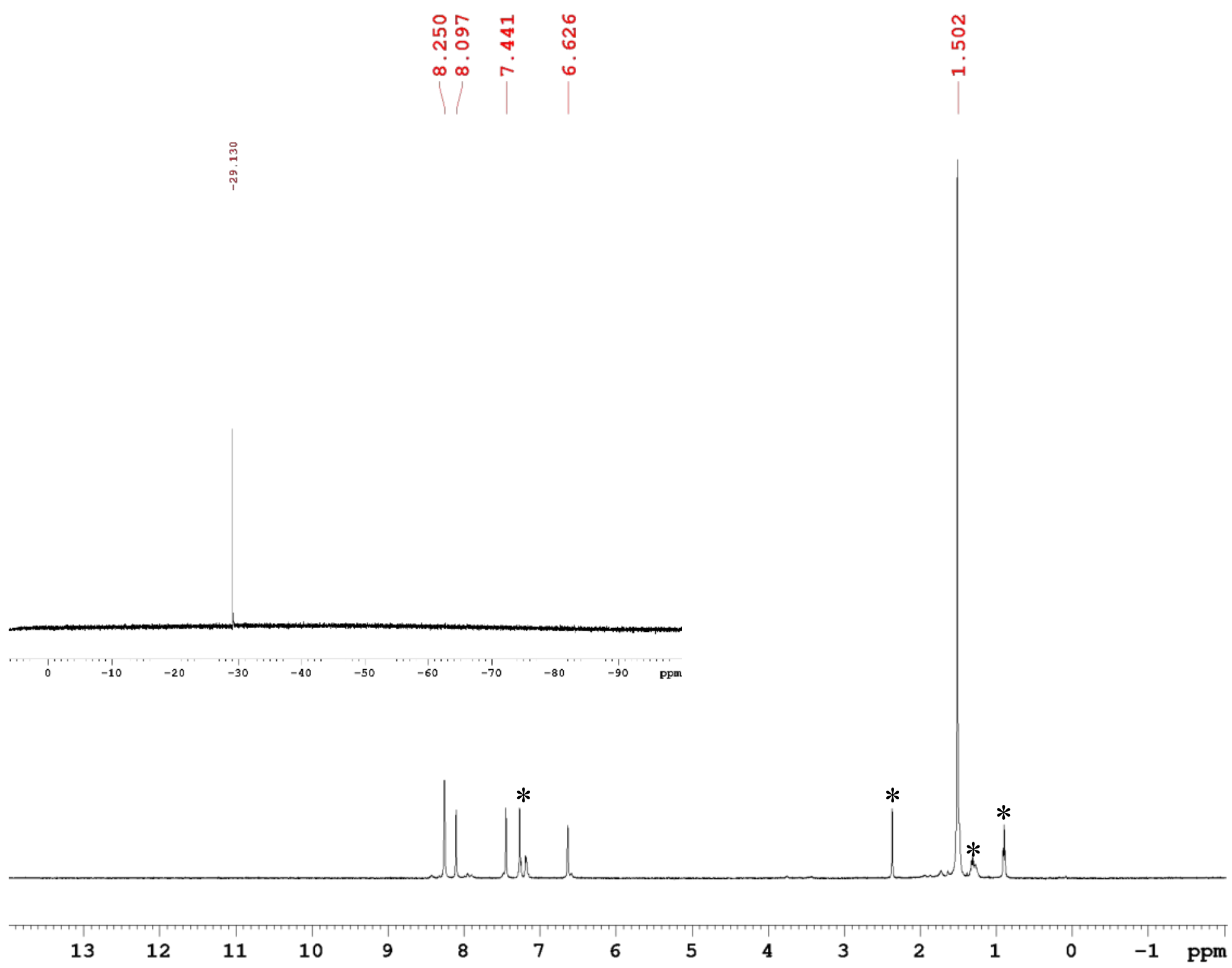

Figure S8. ^{1}H NMR spectrum of $Cz^{tBu}(Pyr^{H})_2NiCF_3$ in $CDCl_3$. Inset: ^{19}F NMR. * solvent residual peaks.

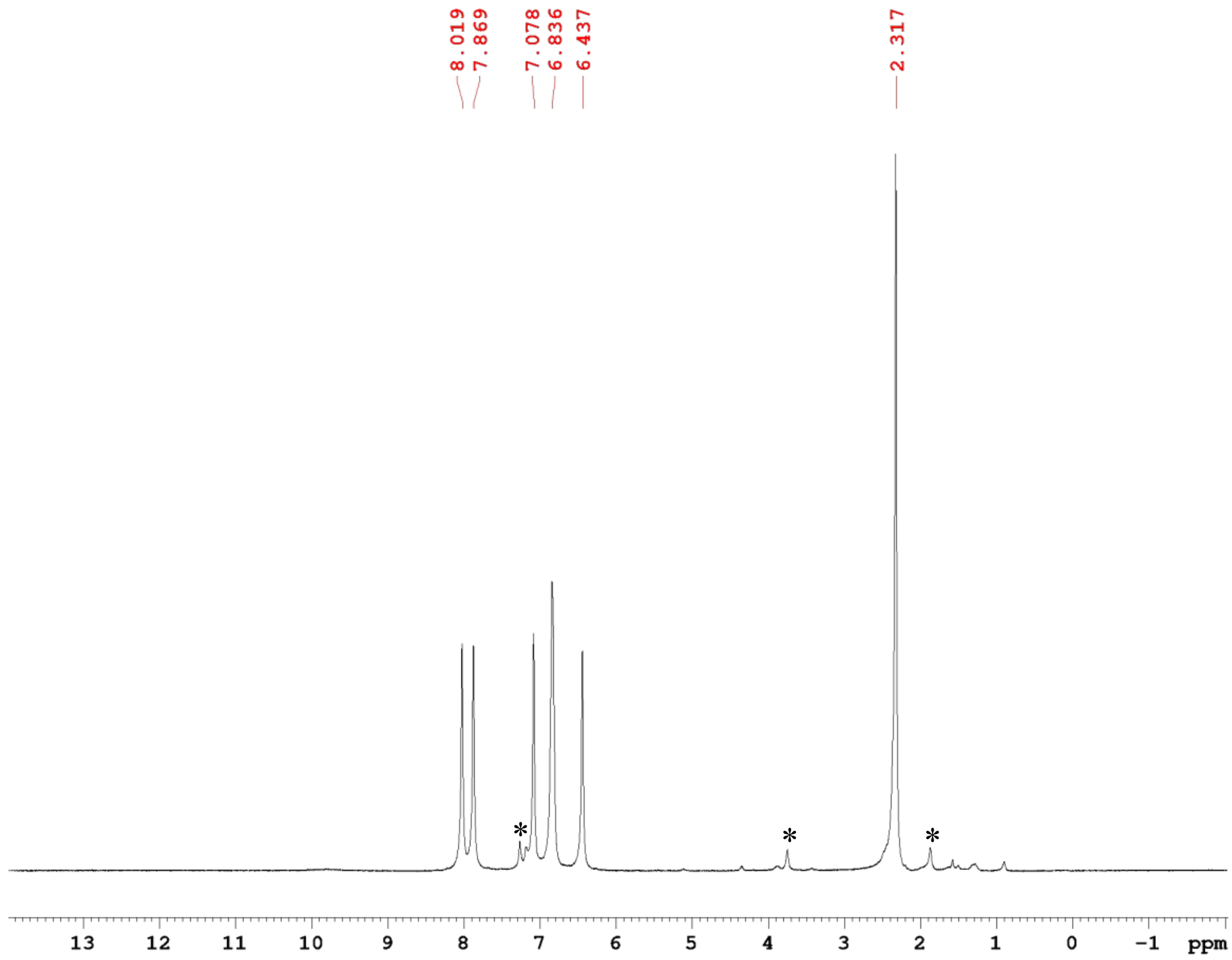

Figure S9. ^{1}H NMR spectrum of $Dpa^{Me}(Pyr^{H})_2NiCl$ in $CDCl_3$.* solvent residual peaks.

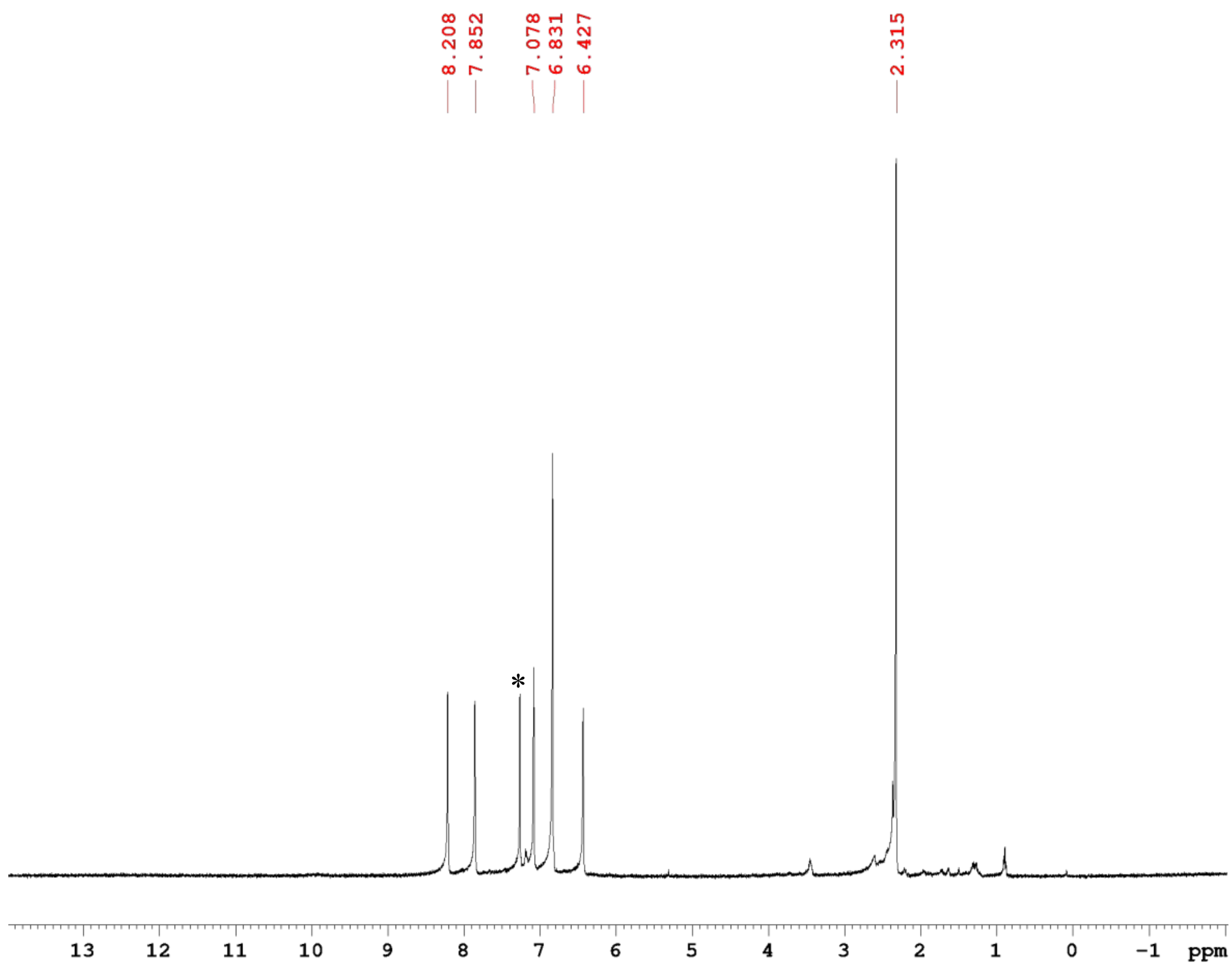

Figure S10. ^{1}H NMR spectrum of $Dpa^{Me}(Pyr^{H})_2NiBr$ in $CDCl_3$. * solvent residual peaks.

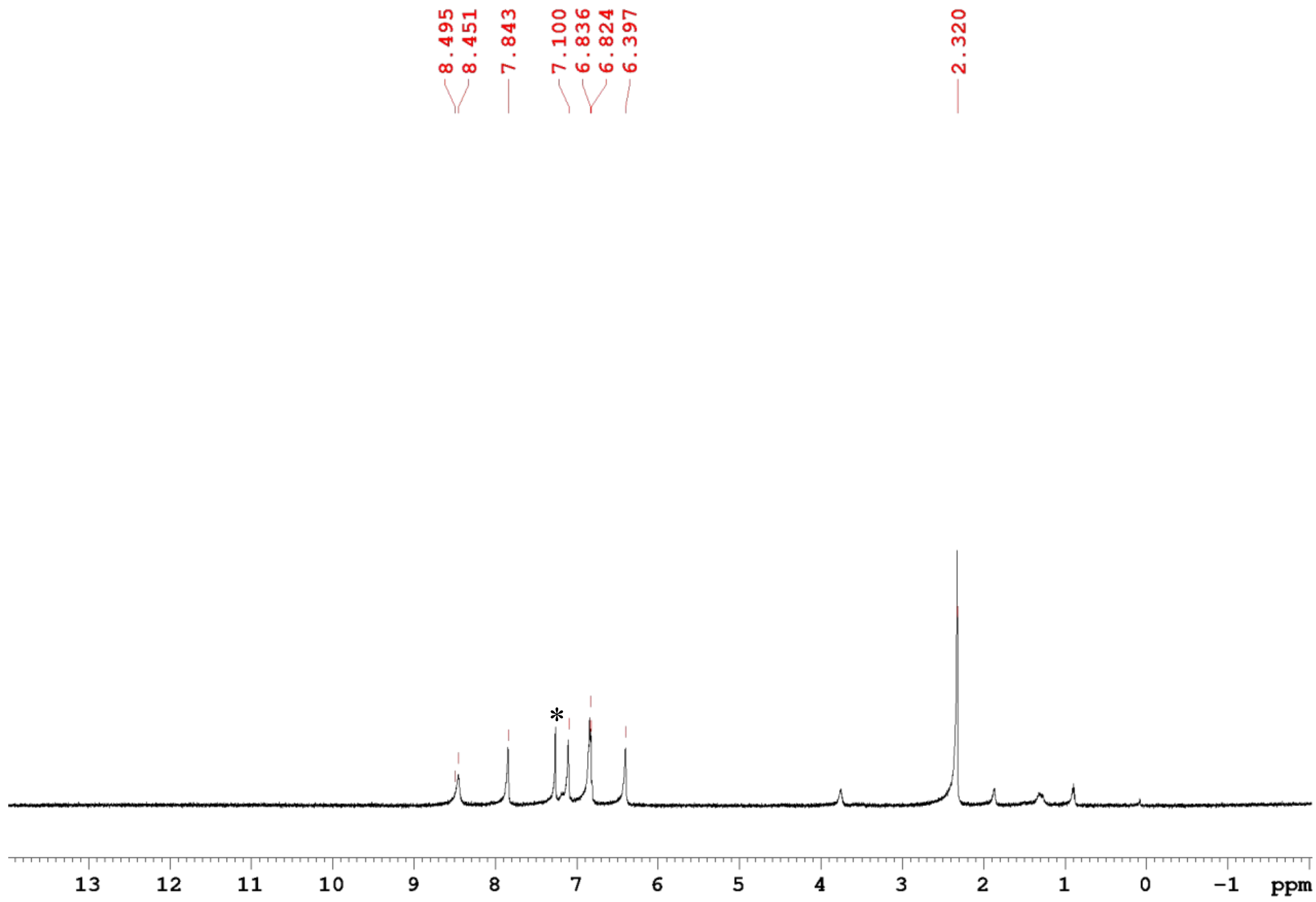

Figure S11. ^{1}H NMR spectrum of $Dpa^{Me}(Pyr^{H})_2NiI$ in $CDCl_3$. * solvent residual peaks.

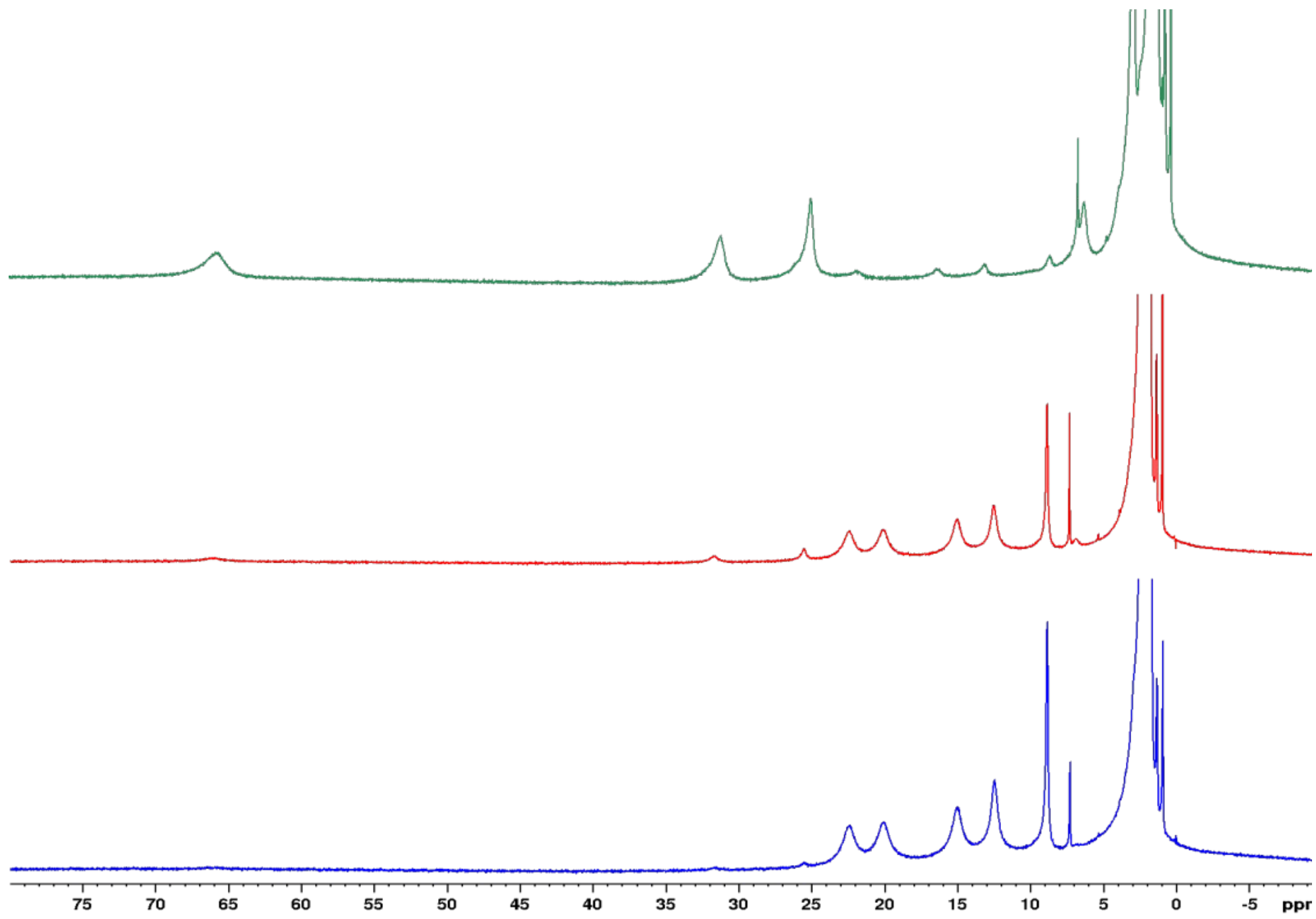

Figure S12. ^{1}H NMR spectroscopic monitoring of the decomposition of *$Cz^{tBu}(Pyr^{H})_2NiBr$* in $CDCl_3$. Overlay of ^{1}H NMR spectra (500 MHz, in C_6D_6) recorded at different time points. Each spectrum represents a time point of 0h (–), 1h (–), 4h (–).

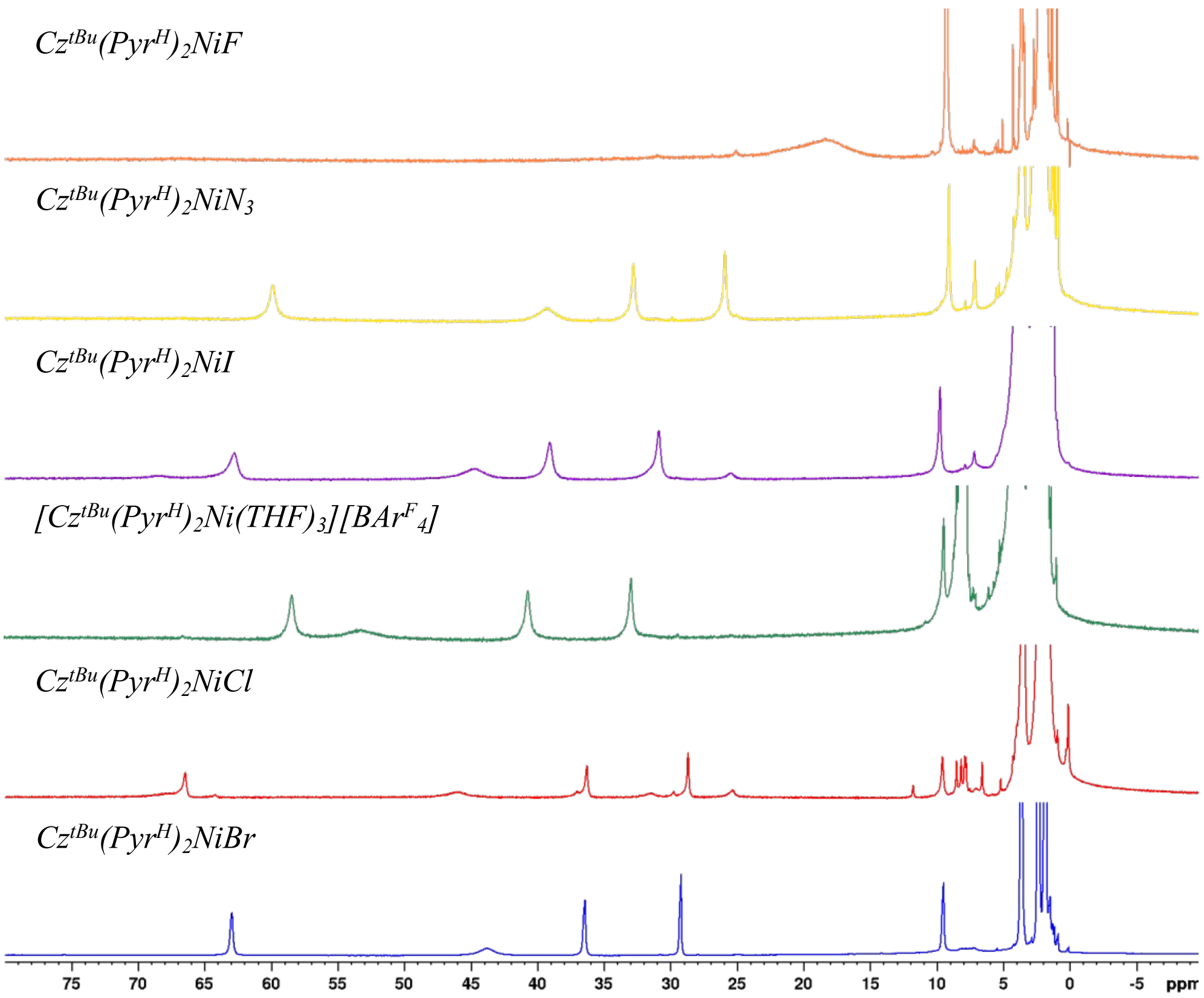

Figure S13. Overlay of ^{1}H NMR spectra (500 MHz, in THF-d_8) of $Cz^{tBu}(Pyr^H)_2NiX$.

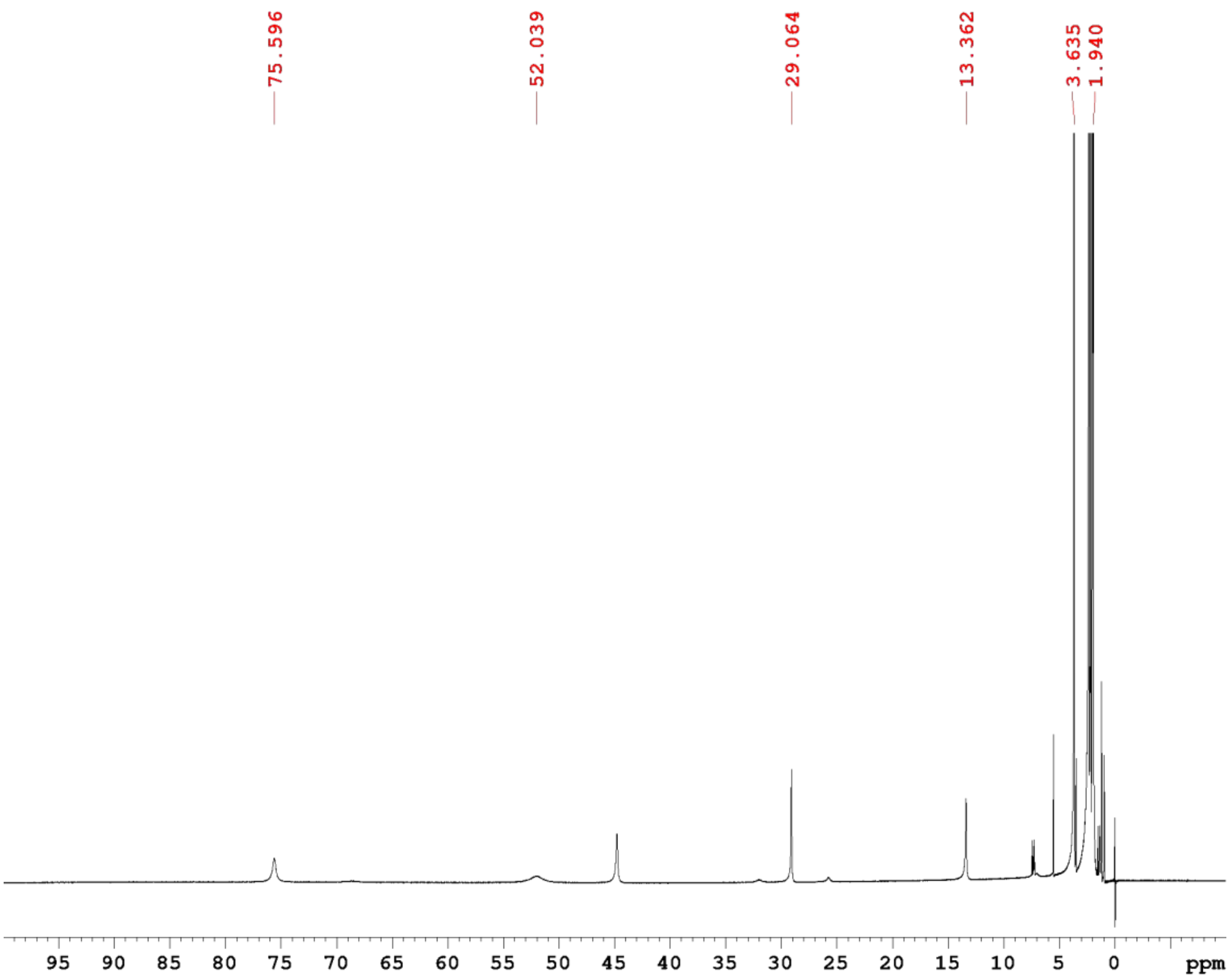

Figure S14. ^{1}H NMR spectrum of $Cz^{tBu}(Pyr^H)_2NiBr$ in CD_3CN.

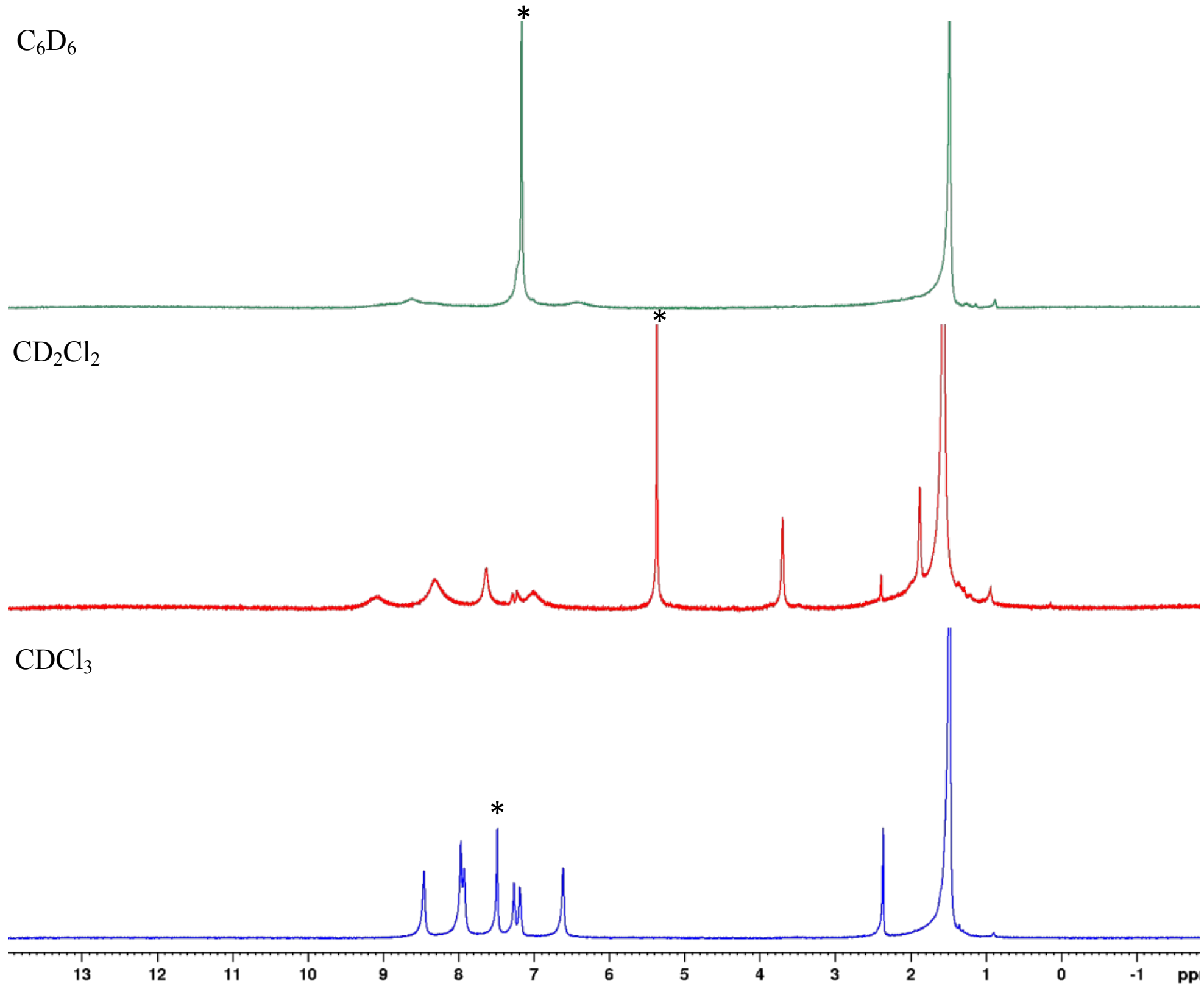

Figure S15. Overlay of ^{1}H NMR spectra of $Cz^{tBu}(Pyr^{H})_2NiF$ in different D-solvents. * solvent residual peaks.

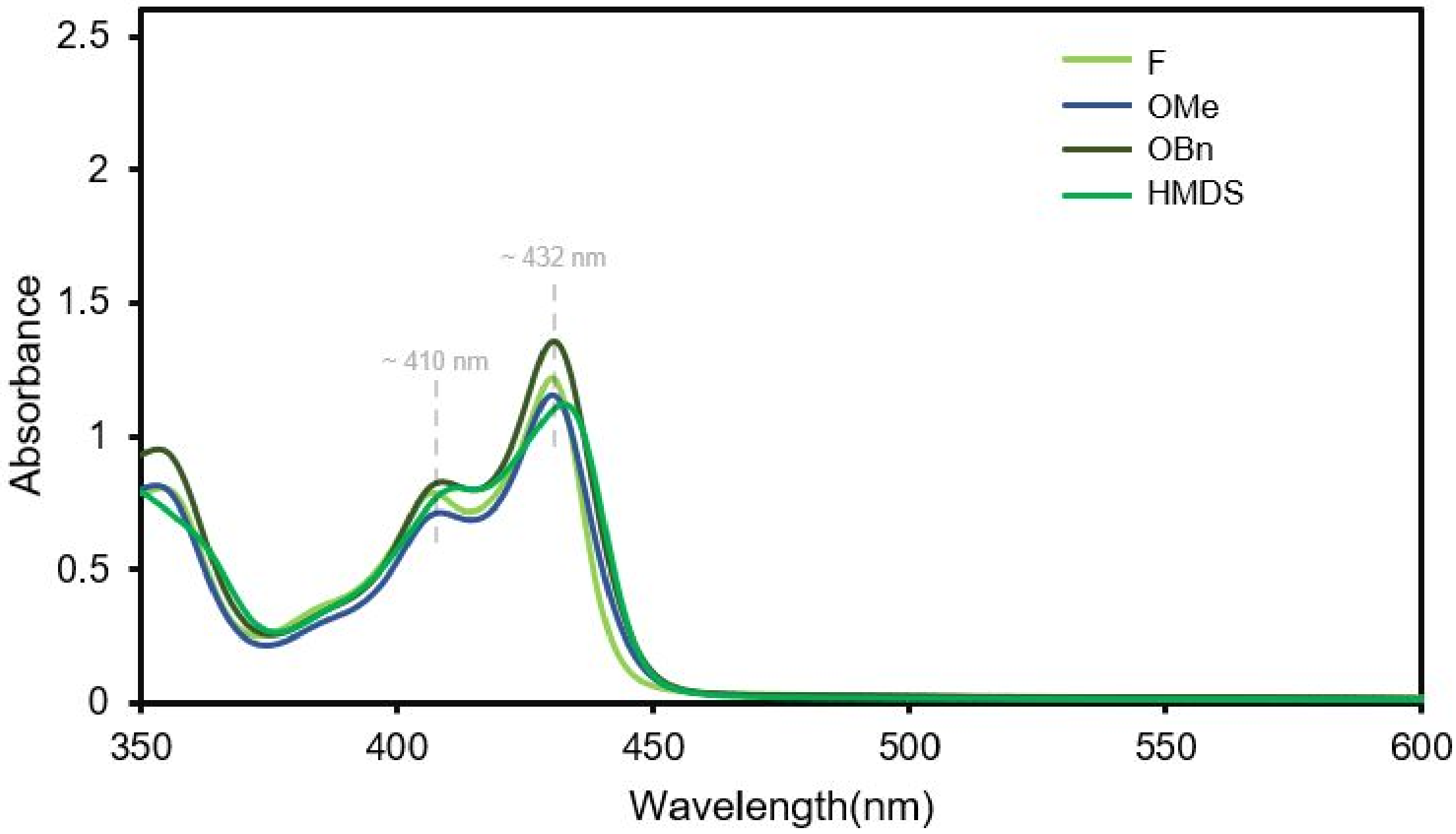

Figure S16. Electronic absorption spectra of 0.1 mM $Cz^{tBu}(Pyr^{H})_2NiR$ (R = F, OMe, OBn, HMDS) in THF.

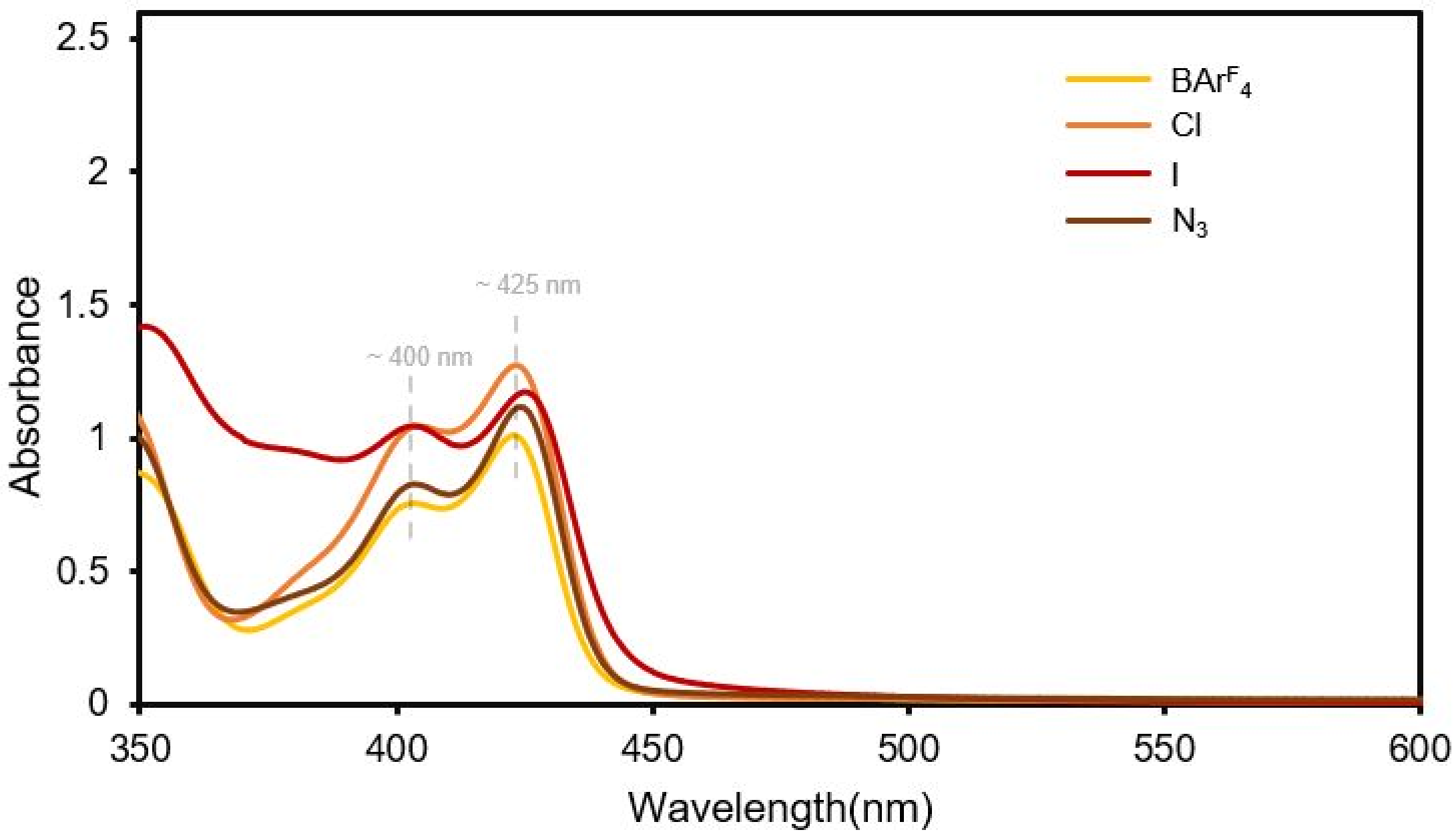

Figure S17. Electronic absorption spectra of 0.1 mM $[Cz^{tBu}(Pyr^{H})_2Ni(THF)_3]X$ (X = BAr^F_4, Cl, I, N_3) in THF.

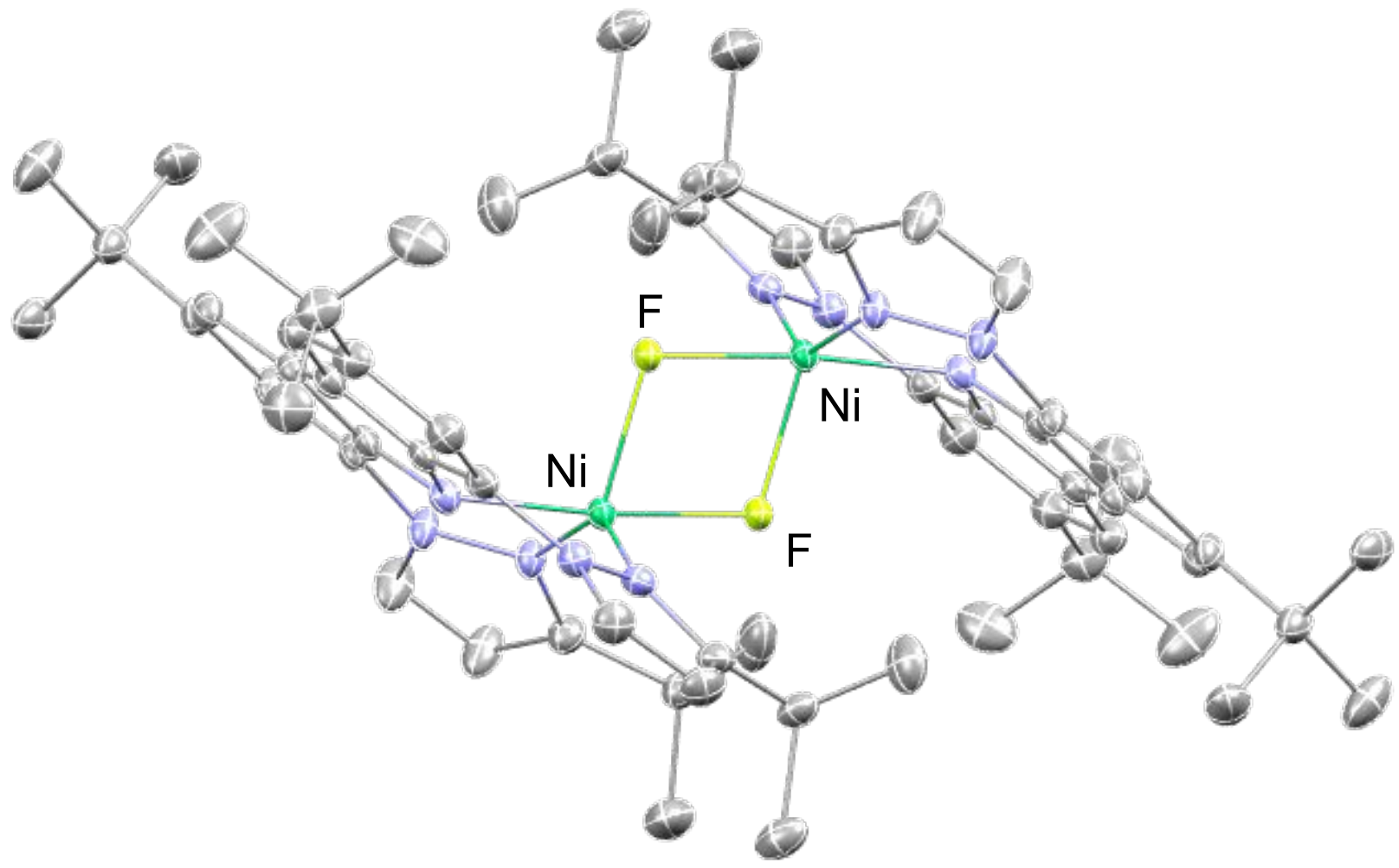

Figure S18. Molecular structure of $[Cz^{tBu}(Pyr^{iPr})_2Ni(\mu\text{-}F)]_2$. Color code: N, blue; C, gray; Ni, teal; F, yellow-green. Hydrogen atoms are omitted for clarity

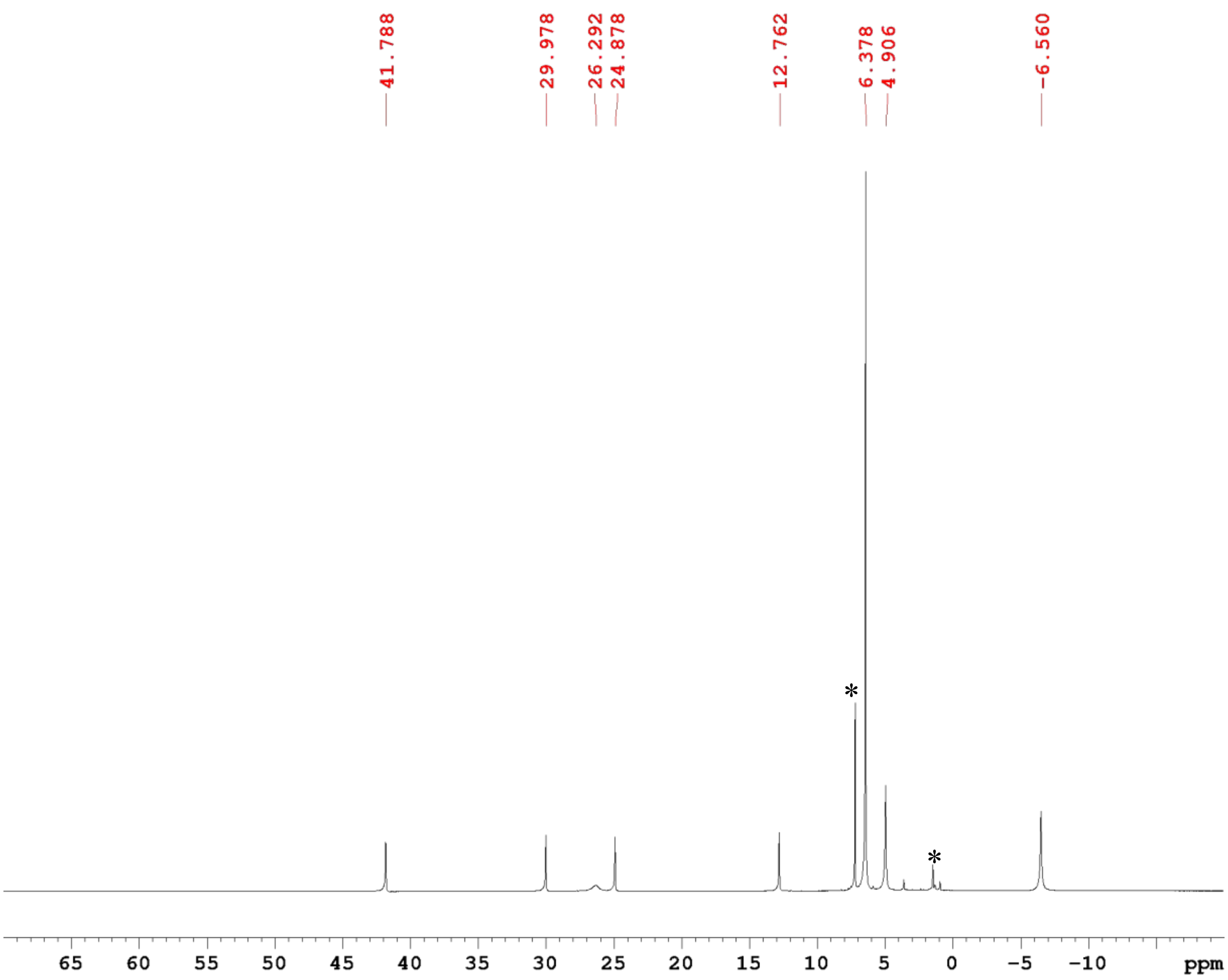

Figure S19. ^{1}H NMR spectrum of $[Cz^{tBu}(Pyr^{iPr})_2Ni(\mu\text{-}F)]_2$ in C_6D_6. * solvent residual peaks.

References

(1) International Organization of Legal Metrology. Standard Solutions Reproducing the Conductivity of Electrolytes. OIML: Paris, 1981.
(2) Macdonald, J. R., Ed. *Impedance Spectroscopy: Emphasizing Solid Materials and Systems*; Wiley, 1987.
(3) Bard, A. J.; Faulkner, L. R. *Electrochemical Methods: Fundamentals and Applications*; Wiley, 2001.
(4) (a) Apex3 v2016.9-0, APEX5 v2023.9-2; Bruker AXS Inc.: Madison, WI, 2016/2023. (b) SAINT V8.37A, SAINT V8.40B; Bruker AXS Inc.: Madison, WI, 2016/2019.
(5) Blessing, R. H. An empirical correction for absorption anisotropy. *Acta Crystallogr A* **1995**, *51 (Pt 1)*, 33–38.
(6) Hubschle, C. B.; Sheldrick, G. M.; Dittrich, B. ShelXle: a Qt graphical user interface for SHELXL. *J Appl Crystallogr* **2011**, *44* (Pt 6), 1281–1284.
(7) Sheldrick, G. M. SHELXT - integrated space-group and crystal-structure determination. *Acta Crystallogr A Found Adv* **2015**, *71* (Pt 1), 3–8.
(8) Sheldrick, G. M. A short history of SHELX. *Acta Crystallogr A* **2008**, *64* (Pt 1), 112–122.
